# Comprehensive analysis of the carboxylesterase gene reveals that *NtCXE22* regulates axillary bud growth through strigolactone metabolism in tobacco

**DOI:** 10.3389/fpls.2022.1019538

**Published:** 2022-12-12

**Authors:** Lin Wang, Xiaodong Xie, Yalong Xu, Zefeng Li, Guoyun Xu, Lingtong Cheng, Jun Yang, Lei Li, Wenxuan Pu, Peijian Cao

**Affiliations:** ^1^ State Key Laboratory of Protein and Plant Gene Research, School of Life Sciences and School of Advanced Agricultural Sciences, Peking University, Beijing, China; ^2^ China Tobacco Gene Research Center, Zhengzhou Tobacco Research Institute of China National Tobacco Corporation (CNTC), Zhengzhou, China; ^3^ Technology Center, China Tobacco Hunan Industrial Co., Ltd., Changsha, China

**Keywords:** carboxylesterase, tobacco, differential expression, axillary bud, strigolactone, NtCXE22

## Abstract

Carboxylesterases (CXE) are a class of hydrolytic enzymes with α/β-folding domains that play a vital role in plant growth, development, stress response, and activation of herbicide-active substances. In this study, 49 *Nicotiana tabacum* L. *CXE* genes (NtCXEs) were identified using a sequence homology search. The basic characteristics, phylogenetic evolution, gene structure, subcellular location, promoter *cis*-elements, and gene expression patterns of the CXE family were systematically analyzed. RNA-seq data and quantitative real-time PCR showed that the expression level of CXEs was associated with various stressors and hormones; gene expression levels were significantly different among the eight tissues examined and at different developmental periods. As a new class of hormones, strigolactones (SLs) are released from the roots of plants and can control the germination of axillary buds.*NtCXE7*, *NtCXE9*, *NtCXE22*, and *NtCXE24* were homologous to *Arabidopsis* SLs hydrolase *AtCXE15*, and changes in their expression levels were induced by topping and by GR24 (a synthetic analogue of strigolactone). Further examination revealed that *NtCXE22*-mutant (*ntcxe22*) plants generated by CRISPR-Cas9 technology had shorter bud outgrowth with lower SLs content. Validation of *NtCXE22* was also performed in *NtCCD8*-OE plants (with fewer axillary buds) and in *ntccd8* mutant plants (with more axillary buds). The results suggest that *NtCXE22* may act as an efficient SLs hydrolase and affects axillary bud development, thereby providing a feasible method for manipulating endogenous SLs in crops and ornamental plants.

## Introduction

Carboxylesterases (CXEs) are a class of hydrolytic enzymes with α/β-folded domains that are found in many animals, plants, and microorganisms, which can influence the hydrolysis of esters and amides (Hatfield et al., 2016). The active sites of CXEs include nucleophilic serine, acidic amino acids (arginine or glutamic acid), and histidine (Marshall et al., 2003). Hydrolysis of natural compounds may cause changes in the biological activity and transport of CXEs, which play important roles in plants. At present, 20 *CXE* genes have been identified in *Arabidopsis thaliana* (Marshall et al., 2003), 16 in *Malus domestica* (Schaffer et al., 2007), 33 in *Prunus persica* (Cao et al., 2019), and 72 in *Gossypium barbadense* (Rui et al., 2022). Plant CXE isoenzymes are found in multiple organs, at various developmental stages, and in various parts of cells (Nomura et al., 2015; Abdel-Daim et al., 2018). The expression of *CXE* genes in plants shows certain tissue specificity (Kamatham et al., 2017). For example, among the 20 *AtCXE* genes identified in *A. thaliana*, *AtCXE13* is only expressed in flowers and fruits, whereas *AtCXE1* is expressed in multiple organs but not in leaves, while other genes are expressed in all plant tissues (Marshall et al., 2003). Furthermore, *CXE* genes are constitutively expressed in plants. The expression of *MdCXE1* is low in the early stages of fruit development but increases sharply after 146 d of flowering (Schaffer et al., 2007). Expression is induced by hormones and pathogens, and in *Vitis flexuosa*, infection with *Botrytis cinerea* upregulates *VfCXE12827*, *VfCXE5585*, and *VfCXE13132* (Islam and Yun, 2016).

Plant CXE proteins have extensive substrate catalytic activities and take part in plant growth and development, secondary metabolism, and biological stress response (Mindrebo et al., 2016). *CXE* genes are also involved in ester metabolism. *MdCXE1* may affect apple flavor by hydrolyzing the 4-methyl umbelliferyl ester substrates in apple fruit at harvest maturity (Souleyre et al., 2011), and Di-n-butyl phthalate (DnBP), commonly used as a plasticizer, is easily absorbed by plants and contributes to the metabolism of rice (Zhu et al., 2019). The expression of *PpCXE1* is related to the catabolic activity of volatile acetate in peach fruits (Cao et al., 2019). CXEs also participate in the regulation of plant tolerance to both biotic and abiotic stresses. For example, *GBCXE49* regulates the tolerance of cotton to alkali stress (Rui et al., 2022); *AtCXE8* enhances plant resistance to gray mold, with the knockout of this gene increasing plant susceptibility (Lee et al., 2013); and *NbCXE* is a novel resistance-related gene that inhibits the accumulation of tobacco mosaic virus (TMV) in tobacco plants (Guo and Wong, 2020). CXEs participate in the activation of plant hormone-signaling substances, regulating IAA metabolism in immature maize endosperm tissues (Kowalczyk et al., 2003). *CXE* genes also regulate strigolactones (SLs) metabolism in *A. thaliana* (Xu et al., 2021) and are involved in herbicide activation. For example, in *A. thaliana*, *AtCXE12* shares the properties of the hydrolytic herbicide precursor methyl-2, 4-dichlorophenoxyacetic acid (Gershater et al., 2007a). Jasmonic acid (JA) seed treatment also influences the expression level of *CXE* genes and promotes the detoxification of mustard seed insecticides (Sharma et al., 2018).

SLs and their derivatives are novel plant hormones derived from β-carotene, which play a crucial role in axillary bud outgrowth (Luo et al., 2019), root elongation (Sun et al., 2021), abiotic stress response (Marzec et al., 2020), and plant-fungi symbiosis (Akiyama et al., 2005). For example, the interference of SLs synthesis gene *CCD8* results in increased branching of potato plants, and its gene editing was found to increase the branching of grapevines (Pasare et al., 2013; Ren et al., 2020). At present, the identified plant endogenous (natural) SLs contain a tricyclic lactone (ABC ring) and monocyclic lactone linked together by an enol ether bond (Yoneyama et al., 2018). The sensory mechanism of SLs is characteristic compared with other phytohormones, as the SLs receptor is an α/β hydrolase folding protein, which is regulated by ligand binding ability and hydrolysability (Toh et al., 2015; Seto et al., 2019). *ShHTL7*, a SLs receptor, enhances binding ability, having a large binding-pocket volume (Chen et al., 2021). Another SLs receptor, *D14*, is a member of the hydrolase family with α/β-folding characteristics, but its binding effect is greater than that of hydrolysis and may not be the key mechanism in SLs hydrolysis (Hamiaux et al., 2012; Zhao et al., 2013). In *A. thaliana*, *AtCXE15* and its homologues have been identified as highly hydrolytic enzymes for SLs; *AtCXE15* catalyzes the hydrolysis of various SLs analogs, and overexpression of *AtCXE15* induces bud branching by SLs (Xu et al., 2021). Ectopic expression of *AtCXE20* in *A. thaliana* and maize also results in increased plant branching and tillering (Roesler et al., 2021).

Tobacco (*Nicotiana tabacum* L.) is an economically important commercial crop and a model plant for genetic studies. Axillary bud germination and lateral branch growth in tobacco plants are also regulated by SLs. However, the *CXE* gene family has not yet been thoroughly evaluated in tobacco. Therefore, in this study, we examine the *CXE* gene family in tobacco and identify those *CXE* genes responsible for environmental stress tolerance and tissue specificity using bioassays. In addition, we verify a *CXE* gene that regulates axillary bud development in tobacco *via* genetic transformation. Our results not only provide a valuable reference for further research into the functional mechanisms of this gene family and the biological functions of CXEs in plant growth and development, but also suggest that CXEs may regulate SLs.

## Materials and methods

### Acquisition and sequence analysis of *NtCXE* family

Tobacco *CXE* (*NtCXE*) genes were found in the tobacco genome database (unpublished) based on the conserved domain (accession PF07859) and protein sequences of *A. thaliana* using “HMMER” software. In addition, the gene length, protein molecular weight, and the theoretical potential of the members of the CXE family of tobacco plants were analyzed using the software “ExPASy” (http://www.expasy.org/tools/). The subcellular localization of 49 *NtCXE* gene family members was carried out using the “Genscript” tool (https://www.genscript.com/psort.html) for prediction. To analyze the evolutionary relationships, the amino acid sequences of *CXE* genes in *Arabidopsis*, tomato, peach, apple and tobacco. were aligned using “CLUSTALX” and “MEGA 7.0” (Kumar et al., 2018).

### Chromosomal location and gene duplication analyses

All the *NtCXE* genes were mapped onto their corresponding chromosomes. “TBtools” (Chen et al., 2020) was adopted to display the positions of chromosome locations and draw the chromosome distribution map of the *NtCXE* genes family. “KaKs_Calculator” was used to calculate the non-synonymous replacement rate (Ka), synonymous replacement rate (Ks), and their ratio (Ka/Ks) (Zhang et al., 2006).

### Gene structure and conserved domain analysis

The “MEME” tool (http://meme.sdsc.edu/meme) was adopted to detect the *NtCXE* conserved motif in members of the gene family. For this, the number of conserved radicals detected was 15, and the length of the motifs was a minimum of six and a maximum of 50 amino acids. The coding sequence (CDS) and genome sequences of the CXE family members were uploaded to the Gene Structure Display Server program (http://gsds.cbi.pku.edu.cn/) to generate an intron-exon structure map.

### *Cis*-acting element analysis and regulatory network prediction

The upstream 3,000-base-pair (bp) sequence of the *CXE* genes were adopted as the promoter region, and the promoter sequence was downloaded from the tobacco genome database. The PlantCare website (http://bioinformatics.psb.ugent.be/webtools/plantcare/html/search_CARE.html) (Lescot et al., 2002) was used to identify *cis*-elements in the promoter regions of the *NtCXE*s. Regulatory elements of promoters were then be classified according to hormones, light, stress, etc. miRNAs downloaded from the miRBase database were used to build the miRNA-*NtCXE* regulatory relationships (http://plantgrn.noble.org/psRNATarget/) (Dai and Zhao, 2011; Kozomara et al., 2019). Transcription factors (TFs) screened from the Plant Transcription Factor Database (PlantTFDB; http://planttfdb.cbi.pku.edu.cn) were used to build the TF-*NtCXE* regulatory relationship network (http://plantgrn.noble.org/psRNATarget/) (Jin et al., 2016), and “Cytoscape” was used to map the regulatory networks (Smoot et al., 2011).

### Plant growth conditions

The common tobacco variety K326 was cultured at the Zhengzhou Tobacco Research Institute in April 2022. Seedlings were grown in a greenhouse with a 14-h light at 28°C/10-h dark at 25°C cycle and a relative humidity of 50-60%. Uniformly growing tobacco (four-week-old seedlings) was screened for hormonal treatment.The tobacco seedlings were planted in 1/2 Hoagland nutrient solution with IAA (10 μM), MeJA (50 μM), ABA (10 μM), SA (10 μM), GA (10 μM), 6-BA (10 μM), GR24 (10 μM), and sucrose (10 mM) for 6 h. Uniformly growing tobacco (six-week-old seedlings) was screened for abiotic stress treatment. Tobacco seedlings were exposed to in 1/2 Hoagland nutrient solution at high temperature (35°C), low temperature (4 °C), salty (150 mM NaCl), dark, cadmium (10 μM), and drought (40% polyethylene glycol, PEG) conditions for 3 d. Roots, stems, leaves, axillary buds, and flowers were subsequently collected during the flowering stage. All collected examples were frozen in liquid nitrogen quickly and stored at -80°C in the refrigerator.

### *NtCXE* gene expression in different tissues exposed to different stress treatments

The transcriptome data was adopted to reveal the expression patterns of *CXE* genes in tobacco in various tissue types and under the different stress conditions. Organizational data including leaves, roots, stems, veins, axillary buds, blades, calluses, and seeds were obtained from the tobacco genome database (unpublished). The sampling method is described in detail in **Supplementary Table 1**. Data on stress, including cold, drought, cadmium (Cd), topping, and CMV and *Phytophthora nicotianae* infection were obtained from the Sequence Read Archive (SRA) (Leinonen et al., 2011; He et al., 2016; Jin et al., 2017; Yang H. et al., 2017; Yang J. K. et al., 2017; Chen et al., 2019). These data were mapped to the tobacco reference genome using “HISAT2.2.1” with default parameters (Kim et al., 2019).

### RNA isolation and expression analysis

Total RNA from each sample was extracted using Trizol reagent. RNA quality and purity were determined using 2% agarose gel electrophoresis and ultraviolet spectrophotometry, respectively. The reverse-transcribed cDNA was synthesized using the Prime Script RT Reagent Kit and stored at -20°C. Primers were designed using the Primer 3.0 online program based on the CDS sequence of *NtCXE* genes for quantitative real-time (qRT)-PCR. An Applied Biosystems CFX96 machine was used for the qRT-PCR with the SYBR qPCR kit (TaKaRa). The tobacco ribosomal protein gene, *L25* (GenBank No. L18908), was used as an internal reference, and three biological replicates were performed (Schmidt and Delaney, 2010). The gene primers used in this study are listed in **Supplementary Table 2**.

### Subcellular localization and GUS staining assay

The Open Reading Frame of the target gene was fused downstream of the PC1300s-GFP vector using *EcroR*I enzyme digestion. The enzymatic digestion product was purified and recombined with the amplified products (ClonExpress-II One Step Cloning Kit). The recombinant plasmid was then transferred into *Agrobacterium tumefaciens* (LBA4404). The monoclonal cells were coated with kanamycin resistant plates and cultured in yeast extract broth liquid medium on a 28 °C shaking table for 2 d. The bacteria were centrifuged at 4,000 rpm/min for 4 min. After supernatant removal, the bacteria were re-suspended in 10 mM MgCl_2_ (including 120 µM AS) suspension, and the OD600 was adjusted to approximately 0.6. The *Agrobacterium* solution was then injected into the lower epidermis (back side) of the tobacco leaves. The injected tobacco plants were then cultured under low light for 2 d and observed using laser confocal microscopy. The empty vector-transformed *A. tumefaciens* was used as a control. The vector map of PC1300s-NtCEX22-GFP was shown in **Supplementary Figure 1**.

The plasmid PBI121 was digested with *BamH*I enzyme and *Sac*I enzyme at 37 °C for 3h. The reaction system included 15 μL of PBI121 plasmid, 1μL of BamHI enzyme, and 1 μL of SacI enzyme. The digested product was analyzed by electrophoresis, recovered, and purified. The promoter sequence of *NtCXE22* was subcloned into the vector pBI121 by clonEZ homologous recombination, and 35S in the vector was replaced. The vector map of proCXE22-GUS was shown in **Supplementary Figure 2**. The proCXE22-GUS vector was then transformed into the tobacco plants (Horsch et al., 1985). The plant materials were placed into β-glucuronidase (GUS) staining solution and then stained for 12 h in the dark at 37°C. After staining, the GUS staining solution was recovered. Plant tissues were immersed in 75% ethanol for decolorization, and after chlorophyll removal, the staining results were photographed for analysis. The gene primers used in this study are listed in **Supplementary Table 2**.

### Gene mutation plasmid construction, plant transformation and mutant analysis

A Cas9/sgRNA vector was constructed as previously described (Gao et al., 2015). According to the mRNA sequence information of *NtCXE22*, two CRISPR target sites of 20 nucleotides were designed to produced small guide RNA (sgRNA). Plasmid Cas9/gRNA was digested by *Bsa*I. enzyme at 37 °C for 4 h. The target site sequence was ligated into pORE-Cas9 binary vector using T4 ligase. The connected carriers were transformed into DH5α competent cells and coated onto lysogeny broth (LB) solid medium. The cells were cultured overnight at 37 °C until positive plaques grew (approximately 16 h). After sequencing, the plasmid was extracted using the OMEGA plasmid extraction kit and transformed into *Agrobacterium tumefaciens* (LBA4404). The vectors were subsequently transformed into tobacco plants using the *A. tumefaciens*-mediated leaf disk method (Horsch et al., 1985). The design method of PCR primers and detection methods of mutation efficiency were carried out accoring to previous literature (Xie et al., 2017).

### Plant tissue safranin O-fast green staining and scanning electron microscope

For plant tissue safranin O-fast green staining, axillary buds were selected as samples and fixed in FAA soution. The sections of samples were rehydrated in BioDewax and put into the safranin O staining solution for 3-8s. The sections were then decolorized and put into plant solid green staining solution for 6-20s. The last, the sections was transparent and sealed for microscope observation.

For the scanning electron microscope, samples (axilalry buds) were quickly taken and fixed with SEM fixation solution for 2h. Then the post-fixation was performed (PBS washing; fixed with OsO4 for 2h; PBS washing). The sample was dehydrated in alcohol and isoamyl acetate. The dehydrated samples were dried and treated with conductive metal coating. Finally, the samples were observed and photographed under a scanning electron microscope.

### Extraction and detection of strigolactone

SL was determined using a plant SL ELISA kit (RJ21771, Shanghai; China). The chemical formula for SL is C_17_H_14_O_5_, the molecular weight is 298.29. Samples were collected from the roots of the wild type and *NtCXE22* mutant plants following the manufacturer’s instructions.

### Statistical analyses

Excel 2016, SPSS 26.0 and GraphPad Prism 9.0 software were used for data analysis and visualization. All treatments and sample assays were performed with at least three replicates, and each biological replicate included at least three uniformly grown plants.

## Results

### Genome-wide identification of the *NtCXE* family in *N. tabacum*

**Figure 1:**
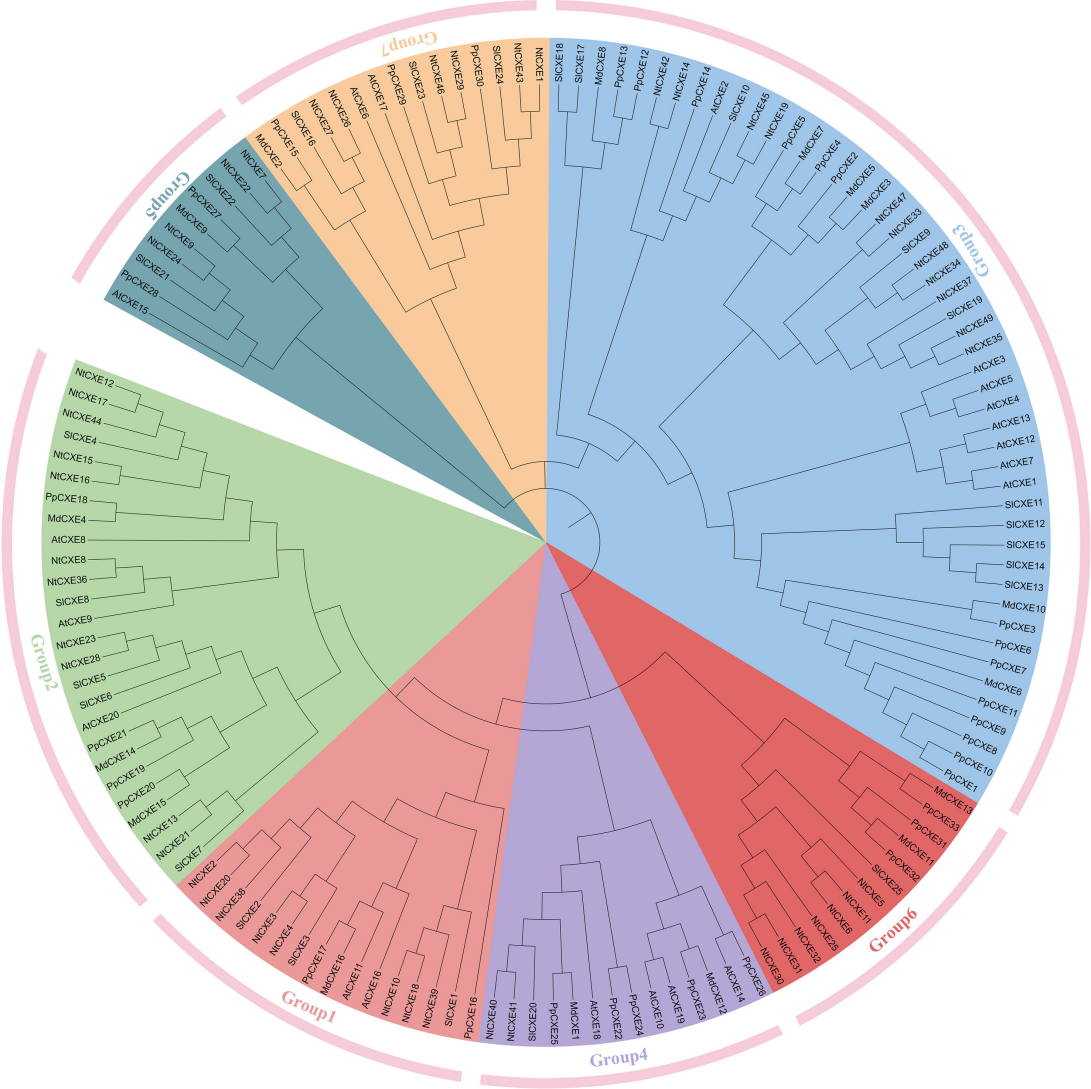
Phylogenetic analysis of carboxylesterase (CXE) families. The Neighbour-Joining (NJ) phylogenetic tree was constructed according to amino acid sequences of *CXE* genes in *Arabidopsis*, tomato, peach, apple and tobacco by MEGA 7.0. The CXE proteins were divided into seven groups (marked as groups 1-7), distinguished by different colors. The standard value of nodes was derived using bootstrapping, with 1000 replicates.

To identify members of the CXE family in tobacco, gene annotation and the hidden Markov model-based profile of the CXE domain (accession PF07859) were used as query conditions, and 49 *CXE* genes were identified in the *N. tabacum* genome database. To understand the evolutionary relationships of CXEs, a phylogenetic tree was constructed using full-length deduced amino acid sequences from *Arabidopsis*, tobacco, peach, apple and tobacco (**Figure 1**). The 49 tobacco *CXE* genes were divided into seven subfamilies according to their sequence homology. Group three contains 12 tobacco CXE members, accounting for 24% of the entire gene family, and was the subgroup with the largest number of members. The CDS length of the *NtCXE* genes ranged from 411 to 1,479 bp, and the protein length ranged from 136 to 492 amino acids. The protein molecular weights (MWs) of the NtCXE proteins were between 15.55 and 53.98 kDa, with the isoelectric points of members of the *CXE* gene family ranging from 4.58 to 5.93. The predicted locations of the NtCXE proteins in the cell were mostly in the cytoplasm and mitochondria based on subcellular localization prediction. The CDS sequences, physical and chemical properties of the 49 identified *NtCXE* genes are listed in **Supplementary Table 3**.

### Chromosomal locations, duplication, and multiple sequence alignment

**Figure 2:**
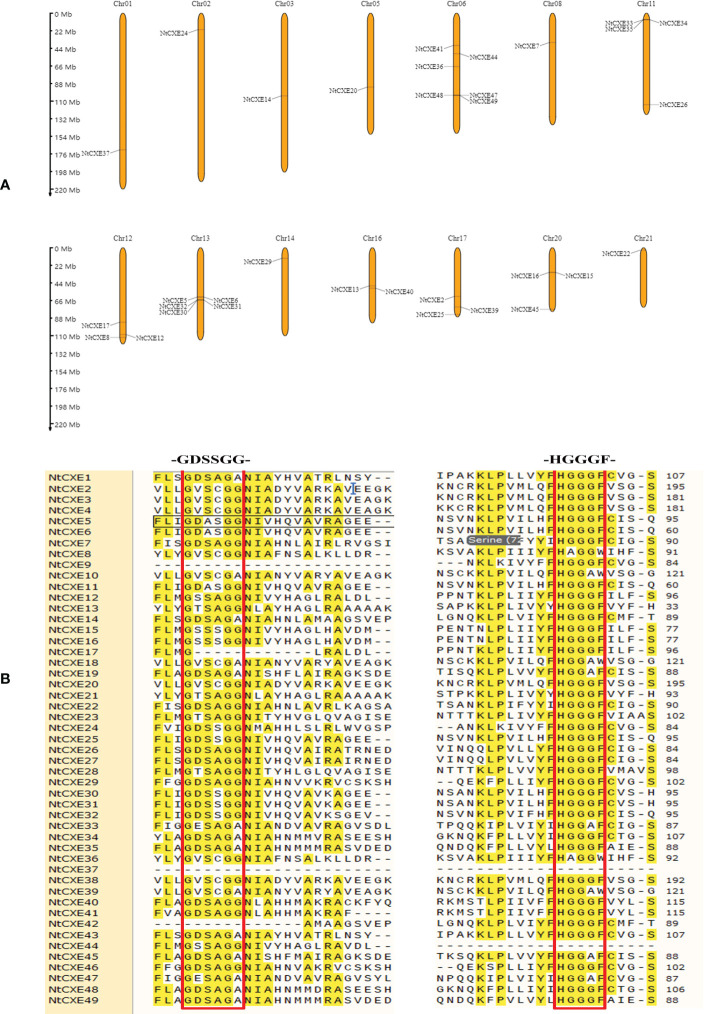
Analysis of genomic location, duplicated gene pairs, and sequence comparison of tobacco NtCXE proteins. **(A)** Chromosomal positions of the *CXE* genes. The chromosomal names are in red and are shown at the top, and the gene names are shown on the chromosome. The length of chromosomes is shown to scale. **(B)** Multiple sequence alignment of conserved domain of NtCXE proteins. The amino acid sequences were aligned using ClustalX.

The chromosome analysis of *NtCXE* genes is presented in **Figure 2A**. We found that 33 *NtCXE* genes were present on the following 14 chromosomes: Chr01, Chr02, Chr03, Chr5, Chr6, Chr8, Chr11, Chr12, Chr13, Chr14, Chr16, Chr17, Chr20, and Chr21. The largest gene cluster (13 members) was observed on Chr6 (**Figure 2A**). There were five *NtCXE*s on Chr13 (*NtCXE5*, *NtCXE6*, *NtCXE30*, *NtCXE31*, and *NtCXE32*), four on Chr11 (*NtCXE26*, *NtCXE33*, *NtCXE34*, and *NtCXE35*), and three on Chr20 (*NtCXE15*, *NtCXE16*, and *NtCXE45*). Chr01, Chr02, Chr03, Chr5, and Chr8 contained the fewest *NtCXE* genes, with only one each. In addition, 16 *NtCXE* genes were not located on a chromosome but were mapped onto certain scaffolds (**Figure 2A**). The nucleotide sequences of the *NtCXE* genes were subsequently compared in a gene replication analysis. A total of 27 gene replication events occurred in the *NtCXE* gene family, including two tandem replication events and 25 segmental replication events. The Ka and Ks values of the gene replication pairs were used to evaluate the factors affecting gene evolution in tobacco. The same type of duplicated gene showed different Ka and Ks distributions; whole genome duplication (WGD)-type repeat gene pairs showed a smaller Ka/Ks ratio, revealing slower sequencing or functionalization over a longer period of time (**Supplementary Table 4**). The CXE family belongs to the α/β sheet hydrolase superfamily, and contain a conserved core-a HGGGF-and-GXSXG-motif-associated with catalysis and degradation (Ueguchi-Tanaka et al., 2005). The *NtCXE* protein sequence alignment showed that this motif was highly conserved (**Figure 2B**). This warrants further study regarding the degradation of *NtCXE*s.

### Gene structure and conserved motif analysis

**Figure 3:**
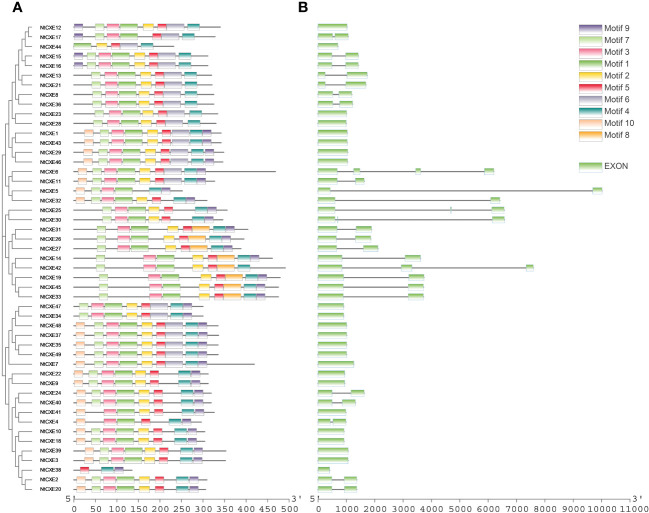
Phylogenetic relationship, gene structures, and protein conserved motif of NtCXE genes. **(A)** Analysis of conserved motif in the amino acid sequences of NtCXEs. The differently colored boxes in the upper right corner represent different conserved motifs with the number 1 to 10. The sequence information corresponding to different motifs is provided in **Supplementary Table 5**. **(B)** Exon-intron structure of NtCXEs in tobacco.

The “MEME” suite tool was chosen to analyze the conserved motifs, 10 of which were identified (**Supplementary Table 5**; **Figure 3A**). Analysis of these genes suggested that the motif of the *NtCXE* gene family had a certain conserved type. Introns are an important component of eukaryotic genes that can participate in the post-transcriptional re-splicing of structural genes. Some introns also participate in the regulation of promoter activity and the activity of response elements contained in promoter introns (Hoshida et al., 2017). The CDS of the *NtCXE* genes was more deeply analyzed with the genome sequences; intron and exon analyses were performed using GSDS 2.0 (**Figure 3B**). The 26 *NtCXE*s were all intron-containing genes, and exons were separated by introns. However, the gene structures were very different, and the number of exons ranged from one to four. Twenty (40.81%) genes had one intron, whereas *NtCXE6* had three introns. Notably, paralogous *NtCXE*s genes shared similar exon/intron distribution rules.

### *Cis*-acting elements and interaction networks of the CXE family

**Figure 4:**
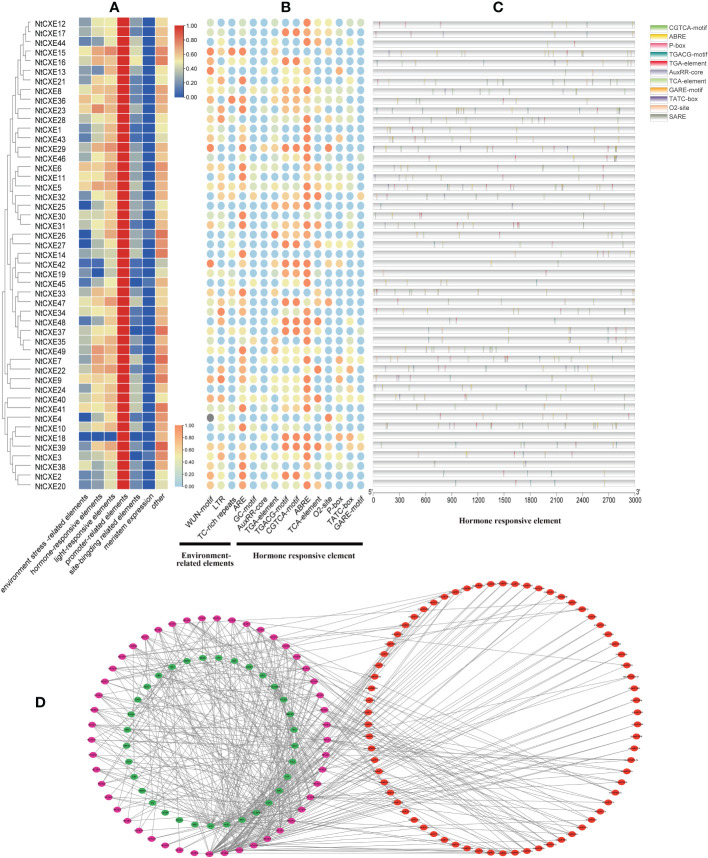
Analysis of promoters and interaction network of the tobacco *NtCXE* genes. **(A)** Predicted *cis*-acting elements divided into six types for each *NtCXE* promoter using PlantCARE software. Heat maps were drawn by TBtools using FPKM mean values. Color represents the gene expression levels (red, high expression level; and blue, low expression level). **(B)** Prediction of the magnitude of elements related to hormone and environmental stress in *NtCXE* promoters. The heat map is drawn in the same way as figure A (orange, high expression level; and blue, low expression level). **(C)** Position, type, and number of hormone-related elements in *NtCXE* promoters using “TBtools”. **(D)** Regulation network of *NtCXE*s in tobacco plants. The purple, green, and red circles denote *NtCXE* genes, transcription factors (TFs), and miRNAs, respectively.

In view of the potential regulatory mechanisms of various *cis*-acting elements in the CXE family, the 3 kb upstream region of the transcription start site was detected, and the putative functions were identified in seven groups. Of these, light-responsive and promoter-related elements were the most abundant (**Figure 4A**). Elements related to the environment include low temperature, defense and stress responsiveness, and anaerobic induction. The plant hormone-responsive category includes auxin, MeJA, abscisic acid, salicylic acid, zein, and gibberellin (**Figure 4B**). Notably, the promoter regions of 44 *NtCXE* members (89.8%) included ABA response elements (ABRE), 35 genes (89.8%) with MeJA response elements, 33 genes (89.8%) with gibberellin response elements, and 30 genes (89.8%) with auxin response elements (**Figure 4C**) (**Supplementary Table 6**). In addition, the promoter regions of the 25 *NtCXE* genes comprised meristem expression related components. Analysis of promoter elements revealed that *NtCXE* genes may take part in many plant growth and developmental processes.

Promoter *cis*-element-binding transcription factors (TFs) can regulate genetic expression. Here, TFs were predicted using “PlantTFDB” and regulatory relationships were displayed using “Cytoscape” (Jin et al., 2016). We predicted 731 TF members binding to the *NtCXE* promoters, divided into 33 TF families including WRKY, TCP, and NAC. Potential miRNA-binding sites for *NtCXE*s were subsequently identified using “PsRNATarget” (Dai and Zhao, 2011). In total, 138 miRNA members from 23 miRNA families were screened, implying their complex and potentially important roles in the regulation of *NtCXE*s. Some of miRNAs have several *NtCXE* targets; nta*-miR167a*, for example, targeted three *NtCXE* genes. In addition, some *NtCXE*s could be targeted by multiple miRNAs; for instance, *CXE2* could be regulated by 20 miRNAs. The regulatory network of *NtCXE*s with transcription factors and miRNAs is shown in **Figure 4D**. Notably, the relationships between *NtCXE*s and TFs/miRNAs require further study. Specific regulatory information is presented in **Supplementary Table 7**.

### Expression patterns of *NtCXE* genes in different tissues

**Figure 5:**
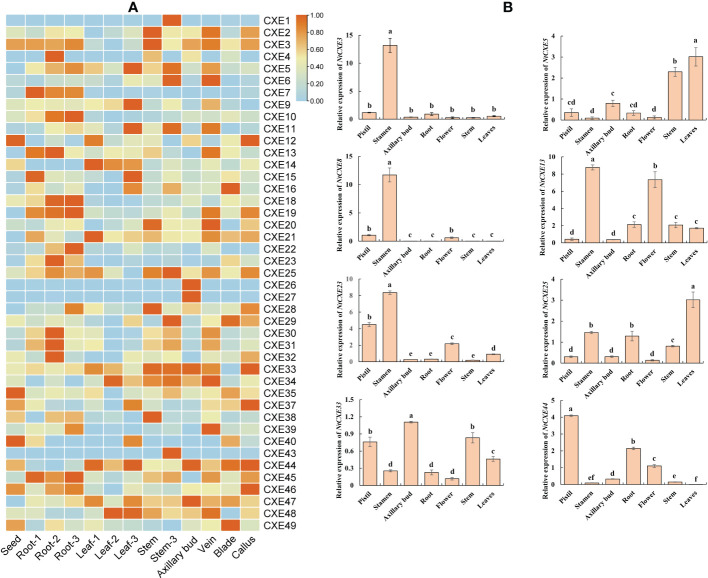
Analysis of tobacco *NtCXE* genes in various plant tissues. **(A)** Expression specificity of all *NtCXE* genes in various plant tissues, some with three developmental stages. ‘1’, ‘2’, and ‘3’ represent the seedling stage, mature stage, and 2 d after topping, respectively. Heat maps were drawn by TBtools using FPKM mean values, scaled by rows. Color represents the gene expression levels (orange, high expression level; and blue, low expression level). **(B)** qRT-PCR analysis of *NtCXE3*, *NtCXE5, NtCXE8, NtCXE13, NtCXE23, NtCXE25, NtCXE33, NtCXE44* in axillary buds, flowers, leaves, roots, terminal buds, and veins. Data are presented as means ± SDs (n = 3). Different letters indicate significant differences between various tissues, based on one-way ANOVA.

To further explore the possible roles of *NtCXE* genes, their expression in eight different tissues (seeds, veins, axillary buds, blades, calluses, roots, stems, and leaves) were screened and analyzed. Of these genes, six were not expressed, and the remaining genes were expressed in five of the screened tissues (**Figure 5A**; **Supplementary Table 8**). *NtCXE35* and *NtCXE49* had higher expression levels in seeds; *NtCXE13* and *NtCXE35* had higher expression levels in seeding roots, *NtCXE21* and *NtCXE35* in seeding leaves, *NtCXE26* and *NtCXE47* in axillary buds, *NtCXE25* and *NtCXE35* in stems, *NtCXE13* and *NtCXE35* in veins, *NtCXE35* and *NtCXE49* in blades, and *NtCXE35* and *NtCXE44* in calluses. Thus, the *NtCXE* genes have distinct expression profiles in different tissues, underlying their potential functions in various physiological processes. In addition, the expression of *NtCXE* genes was significantly affected by the developmental stage (i.e., seedling, maturity, and 2 d after topping). Topping promoted *NtCXE* gene expression in roots but decreased expression in leaves and stems, including *NtCXE5* and *NtCXE28*. Topping increased the expression level of NtCXE7/9/22 in the leaves. *NtCXE* gene expression at maturity was significantly different from that at the seedling stage. For example, *NtCXE38* was not expressed in seedling roots but was expressed in roots at the mature stage, and *NtCXE29* was highly expressed in seeding leaves but was significantly decreased in mature leaves. Eight *NtCXE* genes (*NtCXE 3*, *5*, *8*, *13*, *22*, *25*, *33*, and *44*) were randomly selected to validate the transcriptome results using qRT-PCR analysis, which showed similar expression patterns (**Figure 5B**).

### Expression of *NtCXE* genes under stress

**Figure 6:**
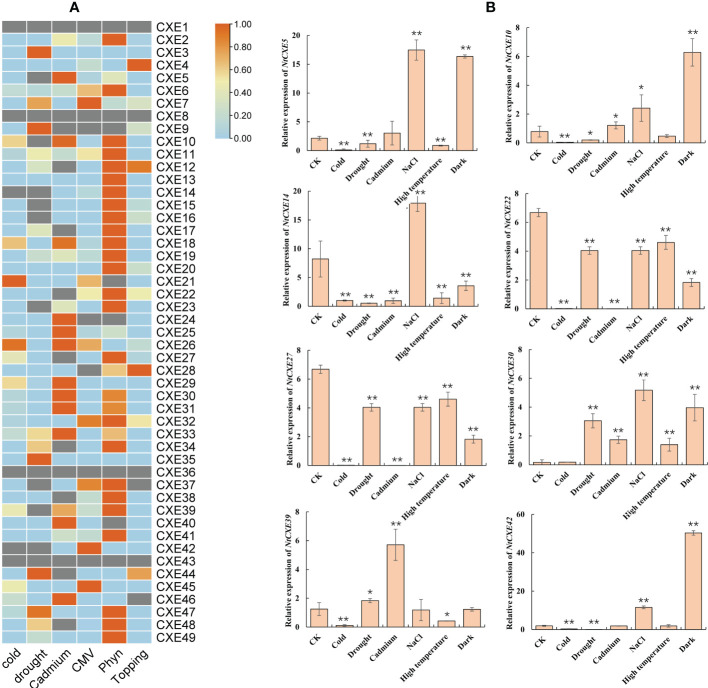
Analysis of tobacco *NtCXE* genes under various stress states. **(A)** Expression profiles of all *NtCXE* genes under cold, cadmium, topping, *P. nicotiana* infection, drought, and cucumber mosaic virus stresses, compared to the control treatment. Heat maps were drawn by TBtools using FPKM mean values, scaled by rows. Color represents the gene expression levels (orange, high expression level; and blue, low expression level). **(B)** qRT-PCR quantitative analysis of *NtCXE5*, *NtCXE10, NtCXE14, NtCXE22, NtCXE27, NtCXE30, NtCXE39, NtCXE42* in response to NaCl, cold, cadmium, drought, high temperature, cold, darkness stressors. Data are presented as means ± SDs (n = 3). *P < 0.05, **P < 0.01 (significant difference between the stress treatment and control, based on Student’s t-test).

To further analyze the *NtCXE* genes involved in stress response, we used publicly available transcriptome data to assess their expression levels (**Supplementary Table 9**). As shown in **Figure 6A**, *NtCXE14* and *NtCXE42* showed a decreasing trend in response to cold stress, while *NtCXE27* showed an increasing trend. *NtCXE7*, *NtCXE47*, and *NtCXE48* were significantly upregulated in response to drought, whereas eight *NtCXE*s were downregulated. *NtCXE5*, *NtCXE25*, and seven other *NtCXE*s were upregulated in response to cadmium, while *NtCXE22*, *NtCXE34*, and seven other *NtCXE*s were downregulated. The expression of *NtCXE6* and *NtCXE7* increased in response to CMV treatment relative to that under normal nutritional conditions, while *NtCXE9*, *NtCXE24*, and *NtCXE28* expression decreased. Sixteen *NtCXE*s showed an increasing trend upon inoculation with *P. nicotianae*, with the expression level of *NtCXE14* increasing by a factor of 5.47 compared to the control. In contrast, the expression levels of six *NtCXE*s decreased upon inoculation with *P. nicotianae*, with *NtCXE9* decreasing by a factor of 6.16 relative to the control. Finally, topping promoted the expression of *NtCXE4* and *NtCXE28* but inhibited the expression of *NtCXE37* and *NtCXE46*. Subsequently, nine *NtCXE* genes (*NtCXE 5*, *10*, *14*, *22*, *27*, *30*, *39*, *42*, and *47*) were randomly selected to validate the transcriptome results using qRT-PCR analysis, which showed similar expression patterns. *CXE* genes were also found to respond to salt, high temperature, and darkness (**Figure 6B**).

### Expression of *NtCXE* genes under the influence plant hormones

**Figure 7:**
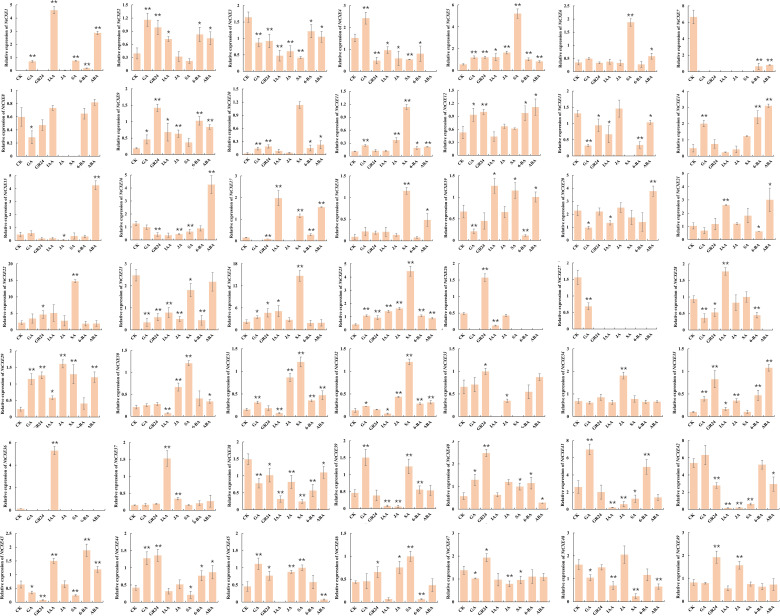
Expression profiles of 49 *NtCXE* genes under diverse hormone treatments. The expression patterns of all *NtCXE* genes in response to ABA, 6-BA, GA, GR24, IAA, SA, and JA were analyzed by qRT-PCR. Seedlings grown under normal conditions were used as controls. Data are presented as means ± SDs (n = 3). *P < 0.05, **P < 0.01 (significant difference between the hormone treatment and control, based on Student’s t-test).

Phytohormones are small-molecule organic substances produced during plant metabolism that move from their production sites to action sites to perform regulatory functions. These hormones play key roles in regulating almost all processes of plant growth and development, and response to environmental stress. To analyze the response of *NtCXE* genes to these hormones, tobacco seedlings were treated with ABA, 6-BA, GA, GR24, IAA, SA, and MeJA, which we found to differentially induce different *CXE* genes. Some genes responded to multiple hormones, such as *NtCXE2*, which responded to ABA, 6-BA, GA, GR24, IAA, SA, and MeJA, while some responded to only one, such as *NtCXE2*, which responded to ABA, and *NtCXE5*, which responded to GA. Notably, for the group-five genes*, NtCXE9/22/24* were all induced by GA, GR24, IAA, SA, and MeJA. *NtCXE7/22/24* were inhibited by ABA and 6-BA(**Figure 7**).

### Identification of the SLs hydrolase genes in tobacco

**Figure 8:**
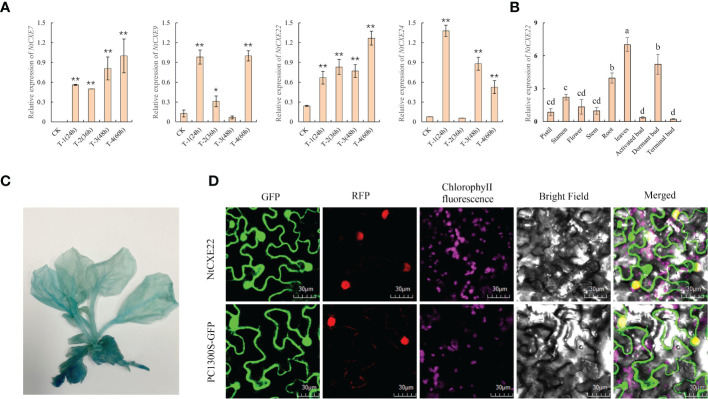
Expression patterns of *NtCXE22* and subcellular localization of the NtCXE22 protein. **(A)** qRT-PCR quantitative analysis of *NtCXE7*, *NtCXE9*, *NtCXE22*, and *NtCXE24* genes at different time points after topping, compared with that before topping. Data are presented as means ± SDs (n = 3). *P < 0.05, **P < 0.01 (significant difference between the topping treatment and control, based on Student’s t-test). **(B)** qRT-PCR quantitative analysis of *NtCXE22* in different tissues. Data are presented as means ± SDs (n = 3). Different letters indicate significant differences between various tissues, based on one-way ANOVA. **(C)** Histochemical analysis of GUS expression driven by proCXE22. **(D)** Subcellular localization of NtCXE22 protein.

In *A. thaliana*, *AtCXE15* participates in hydrolysis of SLs and affects axillary bud development. Through homology comparison, we found that in tobacco, *NtCXE7*, *NtCXE9*, *NtCXE22*, and *NtCXE24* are homologous to *AtCXE15*, being significantly promoted in the axillary buds at different time points after topping (**Figure 8A**). Among them, the expression level of *NtCXE7* and *NtCXE22* was also increased in the roots after topping (**Figure 6A**). According to the induction of GR24 and its highest homology of *AtCXE15*, *NtCXE22* was selected further verify its function in axillary bud development. *NtCXE22* was expressed in different tissues, and its expression level in dormant axillary buds was higher than that in the other two types of axillary bud (**Figure 8B**). The constructed plant expression vector with GUS gene fusion of the *NtCXE22* promoter was used to infect tobacco seedlings *via Agrobacteria*-mediated transient transformation, and GUS staining was adopted to verify the tissue expression pattern of *NtCXE22*. We found that the GUS gene driven by the *NtCXE22* promoter was expressed in all tissues, which was similar to the qRT-PCR results (**Figure 8C**). To determine the subcellular localization of *NtCXE22*, a PC1300s-NtCEX22-GFP construct was introduced into the tobacco leaf protoplasts. As shown in **Figure 8D**, the GFP was predominantly localized in the nucleus and cytoplasm (The original pictures were shown in **Supplementary Figure 3-12**). These results are consistent with the network predictions and confirm the location of NtCXE22 in the cytoplasm (**Figure 8D**).

### Knockout of *NtCXE22* inhibits tiller bud outgrowth in tobacco plants

**Figure 9:**
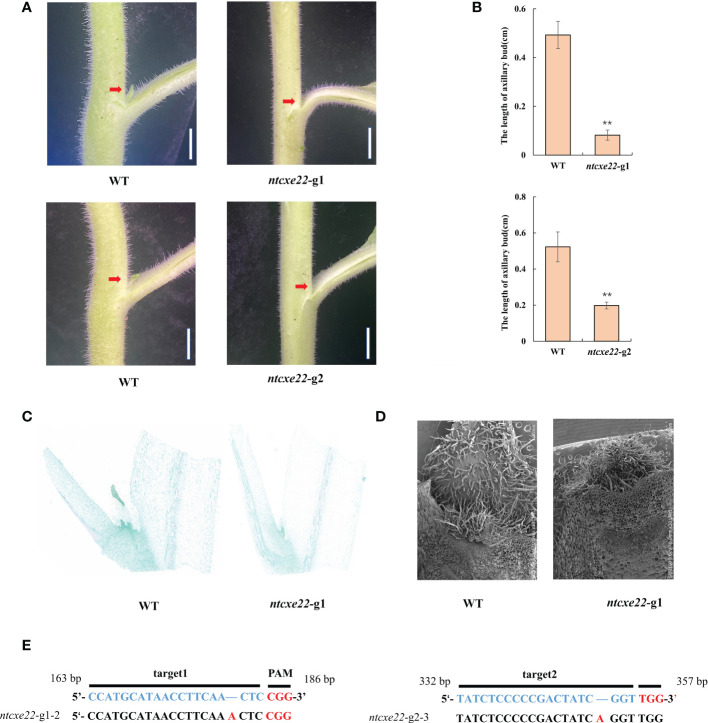
CRISPR/Cas9-mediated gene editing of *NtCXE22* in tobacco. **(A)** Appearance of visible axillary buds in the wild type and *NtCXE22* mutant (*ntcxe22*) tobacco seedings. Scale bar = 1 cm. Phenotypes of the whole plants were shown in **Supplementary Figures 13, 14**. **(B)** Quantitative analysis of axillary bud length in the wild type and *ntcxe22* tobacco seedings. Data are presented as means ± SDs (n = 3). *P < 0.05, **P < 0.01 (significant difference relative to controls based on Student’s t-test). **(C)** Paraffin section of axillary bud in the wild type and *ntcxe22* tobacco seedings. **(D)** Scanning electron micrograph of axillary bud in the wild type and *ntcxe22* tobacco seedings. **(E)** Target locations in *NtCXE22* are marked with blue letters, the protospacer adjacent motif (PAM) with red letters, and mutations in *ntcxe22* -g1 and *ntcxe22* -g2 are also shown.

To further understand the function of *NtCXE22* in axillary buds, targeted *NtCXE22* mutants were built using the CRISPR/Cas9 technology. The two 20 bp target sequences were introduced into a Cas9 vector and transformed into tobacco using *A. tumefaciens*-mediated transformation. Ten T0 transgenic lines were obtained from the two editing sites, which were evaluated for mutants. Among the ten plants, six mutants were identified in this study with a ratio of 60%. *NtCXE22* mutants (*ntcxe22)* resulted in the smaller axillary buds than in the wild type tobacco plants (**Figure 9A**). The axillary bud length of wild-type and *ntcxe22* plants were also quantified in the **Figure 9B**. Sections and electron microscope images of the axillary buds in wild-type and *ntcxe22*-g1 plants are shown in **Figures 9C-D** (The original pictures were shown in **Supplementary Figures 15-18**). Cells in the wild type plants divided more rapidly than those in the *NtCXE* plants. The mutation sites of the two mutated materials (*ntcxe22*-g1-2*, ntcxe22*-g2-3*)* are shown in **Figure 9E**, which were determined to be putative homozygous genotype.

### *NtCXE22* participates in SLs regulation

**Figure 10:**
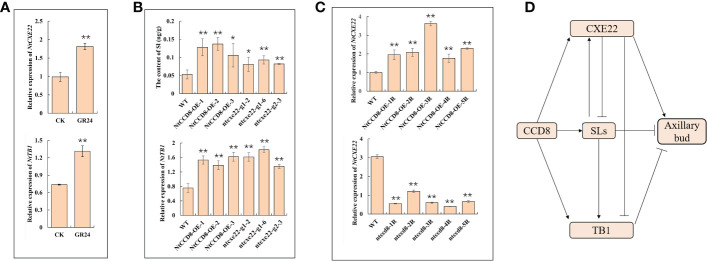
The verification of *NtCXE22* involved in regulating SLs. **(A)** Transcriptional response of *NtCXE22* and *NtTB1* to GR24 (strigolactone analog) by qRT-PCR. **(B)** Expression level of *NtTB1* and the content of SL in the axillary buds and roots of the wild type, *NtCCD8*-OE, and *ntcxe22* plants by qRT-PCR. **(C)** Expression level of *NtCXE22* were monitored in the roots of *NtCCD8* mutant (*ntccd8)* and *NtCCD8*-OE plants by qRT-PCR. **(D)** The regulatory network between the CXE22, CCD8, SL, and TB1. Data are presented as means ± SDs (n = 3). *P < 0.05, **P < 0.01 (significant difference relative to controls based on Student’s t-test).

The SLs-affected *NtCXE22* plants had axillary bud phenotypes similar to those of plants over-expressing *NtCCD8*, a synthetic gene that inhibits the axillary bud development (Pasare et al., 2013; Ren et al., 2020). Therefore, we hypothesized that *NtCXE22* might affect axillary bud development through SLs signaling. *NtTB1*, a SLs downstream target gene, that inhibits axillary bud outgrowth (Braun et al., 2012; Dun et al., 2013). To investigate further, we first monitored the expression levels of *NtCXE22* and *NtTB1* in the axillary buds of tobacco lines exposed to GR24. The exogenous application of GR24 induced an increase in *NtCXE22* and *NtTB1* expression (**Figure 10A**). Moreover, in the *ntcxe22* plants, we found the SL content was increased in the roots, and the expression levels of *NtTB1* was increased in the axillary bud relative to that in the wild type, similar to the phenotypic changes in the *NtCCD8*-overexpression (*NtCCD8*-OE) plants (**Figure 10B**). These results preliminary verify that *NtCXE22* has a regulatory effect on axillary bud development *via* SL catabolism or impaired signaling. Moreover, the expression level of *NtCXE22* was determined in transgenic plants with a distinct axillary bud phenotype. For this, *NtCCD8-OE* plants with smaller axillary buds were cultivated, and CRISPR/Cas9-mediated gene editing of *NtCCD8* (*ccd8*) were cultivated, with more axillary buds than in the wild type plants. The relative expression of *NtCXE22* was increased in the roots of the *NtCCD8*-OE and reduced in the *ntccd8* plants (**Figure 10C**). The regulatory network between the CXE22, CCD8, SL, and TB1 in the tobacco were shown as follow (**Figure 10D**). These results strongly suggest that *NtCXE22* regulates axillary bud development not only by mediating SL signaling but also through other pathways, which require further study.

## Discussion

### Characteristics of the *NtCXE* gene family

CXEs are enzymes with α/β-folding domains that catalyze the hydrolysis of esters and amides and play a role in many physiological reactions in plants (Mindrebo et al., 2016; Lin et al., 2017). The functions of *CXE* genes have been extensively investigated in *Arabidopsis*, cotton, peaches, and other plants, but there are few studies on tobacco, despite it being a model plant (Yang et al., 2008; Cao et al., 2019; Rui et al., 2022). Based on our results, 49 *CXE* genes were identified and characterized in tobacco, which is more than in *Arabidopsis* (20), peach (33), and apple (16) (Schaffer et al., 2007; Mindrebo et al., 2016; Lin et al., 2017). The molecular weight of the *NtCXE* gene family was varied from 15.56 to 53.98 kDa, and most NtCXEs have pI values of <7, which indicates that most NtCXE proteins are acidic. The phylogenetic tree divided *CXE* genes into seven groups, which is similar to other plants. Members of the same subfamily are similar in CDS length, molecular weight, and motifs, suggesting that they may have similar functions.

Gene duplication and subsequent functional divergence are important drivers of genome and species evolution. Tandem replication, genome-wide replication, and fragment replication play major roles in the expansion of individual gene families (Panchy et al., 2016; Kong et al., 2019). In our study, 49 tobacco *CXE* genes were found to be distributed on 14 chromosomes, and two replicas containing five gene clusters were distributed across 13 chromosomes. All of these patterns suggest that gene duplication may benefit gene expansion in the tobacco *CXE* gene family. According to the motif and gene structure analyses, the tobacco *CXE* gene family is relatively conserved. Among them, all members contained motif 4, which can be used to explore the putative origin of *CXE*s. Introns are not directly involved in the proteome, but introns usually contain regulatory elements. Thus, the number and length of introns can affect the protein-coding potential of the genome (Jacob and Smith, 2017; Morgan et al., 2019; Parenteau et al., 2019). Based on previous research, we assume that the long intron of *NtCXE11* may be used to further explore the vital regulatory roles of these genes. The introns and exons of the coding regions of eukaryotic genes control gene transcription and can, therefore, be used to further study the evolution of *CXE*s.

The analysis of promoter *cis*-elements can help understand the tissue specificity and regulatory functions of genes. Numerous environmental stress- and hormonal-response elements are widely distributed, which suggests crucial functions in plant bio/abiotic stress resistance (Rui et al., 2022). Furthermore, TFs, miRNAs, and enzymes can form a complex network that influences plant biological processes (Ibraheem et al., 2010; Chen et al., 2018). TFs interact with *cis*-acting elements on downstream gene promoters to regulate the expression of target genes and induce a series of responses, thereby enhancing plant growth and development (Priest et al., 2009). In our study, accoring to the *cis*-elements, 731 TFs from 33 TF families may be associated with Nt*CXE* gene regulation. miRNAs regulate various biological functions by controlling the expression of target genes (Begum, 2022; Guo et al., 2022). In our study, 138 miRNA members predicated may have regulatory relationships with *NtCXE* genes. Specifically, we found that three *NtCXE*s (*NtCXE2*, *NtCXE38*, and *NtCXE20*) are targeted by miRNA167, which is involved in the regulation of *Arabidopsis* flowering time (Yao et al., 2019). NAC, a well-known flower-development-related TF (Wang J et al., 2022), may also be involved in regulating *NtCXE38* expression. miR156-*NtCXE45*-ERF was another regulation module, and previous studies indicate that both miR156 and ERF are involved in drought tolerance (González-Villagra et al., 2017; Yu et al., 2022). Our interaction network can further contribute to the functional research of *NtCXE*s. Notably, 36 *NtCXE*s can be targeted by miR169, which is involved in plant disease and abiotic stress regulation (Luan et al., 2015; Hanemian et al., 2016), and *NtCXE22* can be targeted by miR482, a miRNA superfamily that is critical for both disease resistance and plant development (Zhang et al., 2022).

### Expression of the *NtCXE* gene family

Plant CXE isoenzymes are found in many plants including in different tissues, organs, and at different developmental stages (Pontier et al., 1998; Ichinose et al., 2001). The *NtCXE* genes are expressed in a wide range of tissues similar to the finding of *AtCXE* genes in *A. thaliana* (Marshall et al., 2003). In apples, *MdCXE1* expression is low during early fruit development and peaks at harvest ripening (Souleyre et al., 2011). In our study, the expression of *NtCXE12* and *NtCXE23* was low in the leaves of seedlings but increased in mature leaves. Topping is an important agronomic procedure in tobacco cultivation, which can promote the development of axillary buds (Wang L et al., 2022) and the accumulation of secondary metabolites in leaves (Zhao et al., 2018). In our results, topping increased the expression of *NtCXE22* in the stems and roots of tobacco and decreased the expression of *NtCXE22* in leaves. We speculate that *NtCXE22* may be involved in the regulation of axillary buds and the secondary metabolism of leaves in tobacco plants. Analysis of *NtCXE* genes expression in different tissues and at different stages should prove helpful in further clarifying the different functions of *NtCXE* genes.

CXEs are highly specific and only act on certain substrates with very high efficiency, whereas other enzymes are able to hydrolyze a wide range of substrates. The functions of *CXE* genes in plants include the activation of plant hormone signaling substances and responses to biotic stresses (Griffiths et al., 2006). The expression of many *CXE* genes is upregulated under abiotic stress, such as alkaline stress (Rui et al., 2022) and *V. flexuosa* infection (Islam and Yun, 2016). In our study, the expression levels of *NtCXE6* and *NtCXE7* were significantly increased after CMV infection, which is consistent with *NbCXE* expression in tobacco infected with TMV (Guo and Wong, 2020). Overexpression of the *AtCXE8* gene in *A. thaliana* enhances resistance to *Botrytis cinerea* (Lee et al., 2013), and in our study, *NtCXE14* expression was significantly increased upon inoculation with *P. nicotianae*. Furthermore, *NtCXE22* was responsive to the cadmium and Phyn infection treatments, and *NtCXE5* was responsive to the drought treatment. These results suggest that *CXE* genes are responsive to a wide range of biotic and abiotic stresses.

Hormone-signaling molecules may control plant physiological processes through conversion between inactive esters and active molecules, and these signaling molecules are released through the selective hydrolysis of esterases (Westfall et al., 2013; Kamatham et al., 2017). Plant CXEs can demethylate inactive methyl salicylate and methyl jasmonate to produce active salicylate and jasmonate (Kumar and Klessig, 2003; Stuhlfelder et al., 2004). In our study, RT-qPCR experiments were performed using multi-hormone treatments, which showed that the expression levels of many *NtCXE*s were significantly enhanced by MeJA and SA, such as *NtCXE5* and *NtCXE22*. CXEs have been reported to control IAA metabolism in immature maize endosperm tissues, and these genes also regulate GA20 glycogroup metabolism in maize (Schneider et al., 1992; Kowalczyk et al., 2003). Similarly, we found that *CXE* gene expression was also induced by IAA, ABA, 6-BA, GA, and GR24. However, the types of hormone-induced genes were inconsistent, indicating that different *CXE* genes participate in different biological processes in response to different hormones, which requires further exploration.

### *NtCXE22* is involved in axillary bud development *via* SL

SLs are newly identified hormones with important applications in agriculture, being notably involved in tillering regulation (Wang et al., 2018). SLs biosynthesis and signaling have been extensively studied in the regulation of axillary bud development (Lin et al., 2009; Vogel et al., 2010; Pasare et al., 2013; Wen et al., 2016; Ren et al., 2020). In particular, *CCD8* (a synthetic SLs gene) mutation caused increased branching in tobacco (Gao et al., 2018), and *CpCCD8* overexpression reduces the branching in the *Arabidopsis CCD8* mutant (Wang et al., 2021). On account of low abundance of SLs, little is known about their inactivation at the catabolic level (Snowden et al., 2005; Arite et al., 2007; Simons et al., 2007). In *Arabidopsis*, *AtCXE15* has been identified as a functionally important SLs hydrolase (Xu et al., 2021), and the ectopic expression of *CXE20* effectively reduces the concentration of free SLs and increases the number of branches (Roesler et al., 2021). These studies indicate a new mechanism of SLs degradation regulation in plants. In our study, *NtCXE22* was identified in tobacco and was homologous with *AtCXE15*. *NtCXE22* is located in the cytoplasm and nucleus, which is consistent with observations in peach and cotton (Cao et al., 2019; Rui et al., 2022). SLs are involved in the regulation of apical dominance in plants, and play a direct inhibitory role in branching (Cheng et al., 2013). Our results imply that topping (i.e., removal of apical dominance) increases the expression of the *NtCXE22* gene in roots and leaves, which may be involved in the regulation of SLs degradation. CRISPR*/Cas9*-mediated gene editing of *NtCXE22* (*ntcxe22*) in tobacco also inhibited axillary bud development with increased SLs content, which is consistent with the phenotype of *AtCXE15.* In addition, altered expression levels of *NtCXE22* in *CCD8* (the SLs synthesis gene) overexpression also indirectly confirms its regulatory effect on SLs. CXEs belong to the ABH superfamily, whose members function as carboxylic ester hydrolases of both xenobiotics and endogenous metabolites in plants (Gershater and Edwards, 2007b). We hypothesize that *CXE22* might mediate SLs catabolism and, thereby, affect axillary bud development, which provides the basis for further research into the molecular mechanisms of *CXE* genes in plant growth and development. In addition, as SLs are distributed in both roots and axillary buds (Gomez-Roldan et al., 2008; Xie, 2016), the targeted degradation of SLs content in different tissues by CXE gene can be regarded as a research direction, which can be used to specifically regulate plant architecture or root system.

## Conclusion

In summary, we explored the evolutionary relationships, functional information, and regulatory networks of the *CXE* gene family in tobacco plants. We successfully revealed the details of 49 genes, including gene structures, chromosomes, promoter *cis*-elements, associated transcription factors, and miRNAs. In addition, the expression levels of *CXE* genes in various tissues, under various abotic stresses, and in response to a range of plant hormones were determined. We found that *NtCXE7*, *NtCXE9*, *NtCXE22*, and *NtCXE24* are regulated by topping and GR24. Furthermore, knockout of *NtCXE22* inhibited axillary bud development and increased SLs content and the expression level of *NtTB1*, which was consistent with *NtCCD8* (the SLs synthesis gene) overexpression lines. Overall, our work provides a solid foundation for the functional study of CXE genes as well as new understanding of the regulation of plant architecture.

## Data availability statement

The datasets presented in this article are not readily available because the China tobacco genome database is not publicly available . Requests to access the datasets should be directed to Peijian Cao, peijiancao@163.com.

## Author contributions

LW, WP, and PC conceived the experiments. LW drafted the manuscript. LW and XX conducted the experiments with assistance from JY and GX. YX, ZL, and LC processed and analyzed the data. LL revised the manuscript. All authors contributed to the manuscript and approved the submitted version.
