# Supplementary material for: Comprehensive analysis of the carboxylesterase gene reveals that NtCXE22 regulates axillary bud growth through strigolactone metabolism in tobacco
Source: Front Plant Sci. 2022 Dec 12;13:1019538. doi: 10.3389/fpls.2022.1019538 (PMC9806860; doi:10.3389/fpls.2022.1019538)
Supplement: Supplementary file 1 [file DataSheet_1.pdf]

Supplementary Figures

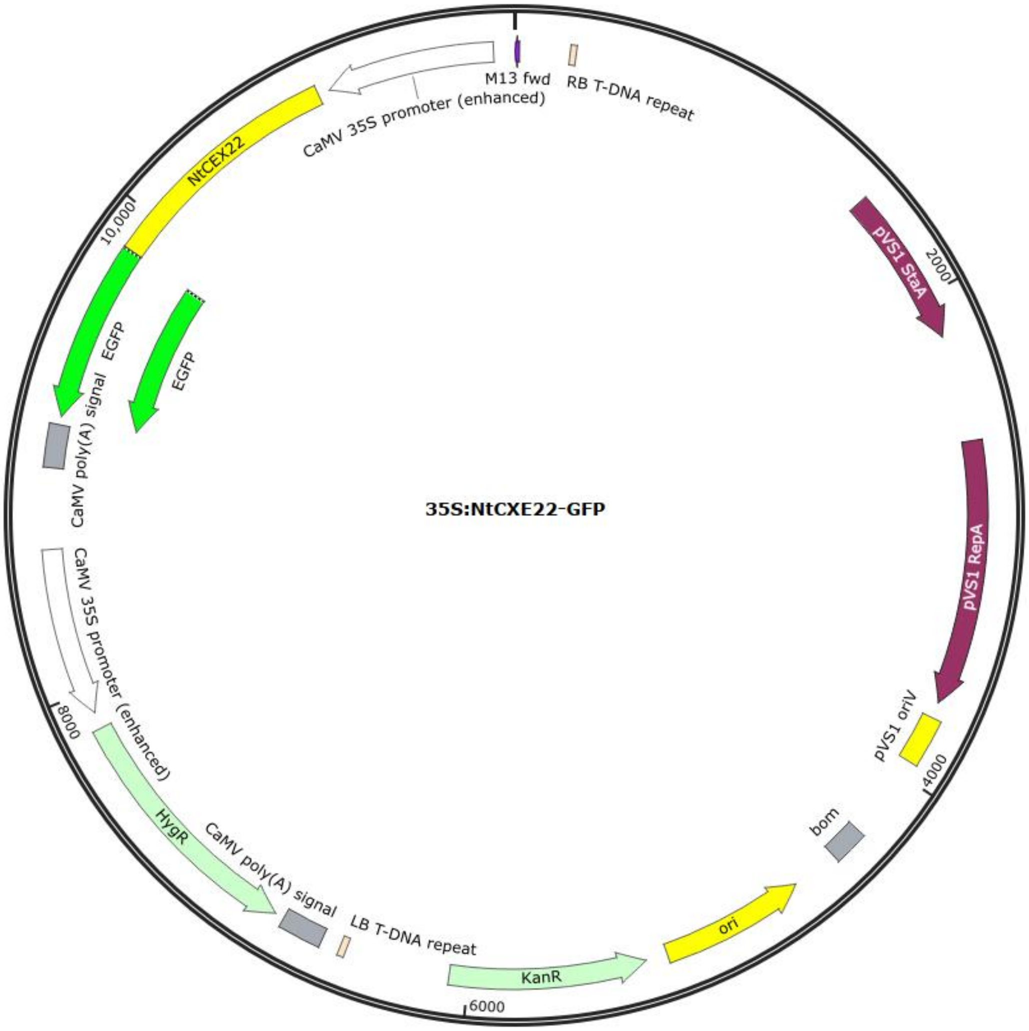

Supplementary Figure 1. The GFP construct and fusion.

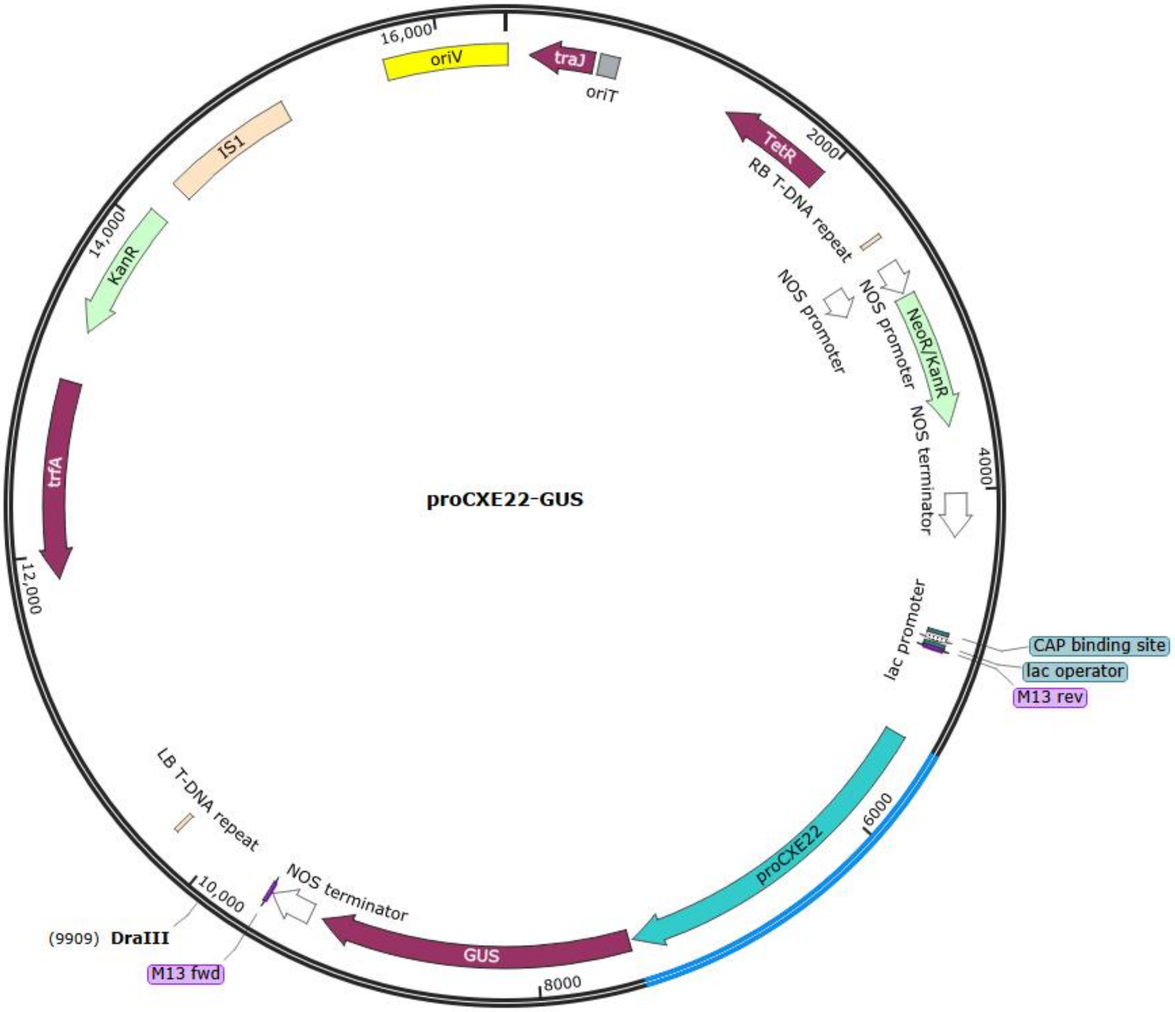

Supplementary Figure 2. The vector map of Pbi121 and generation of *NtCEX22* promoter driving GUS.

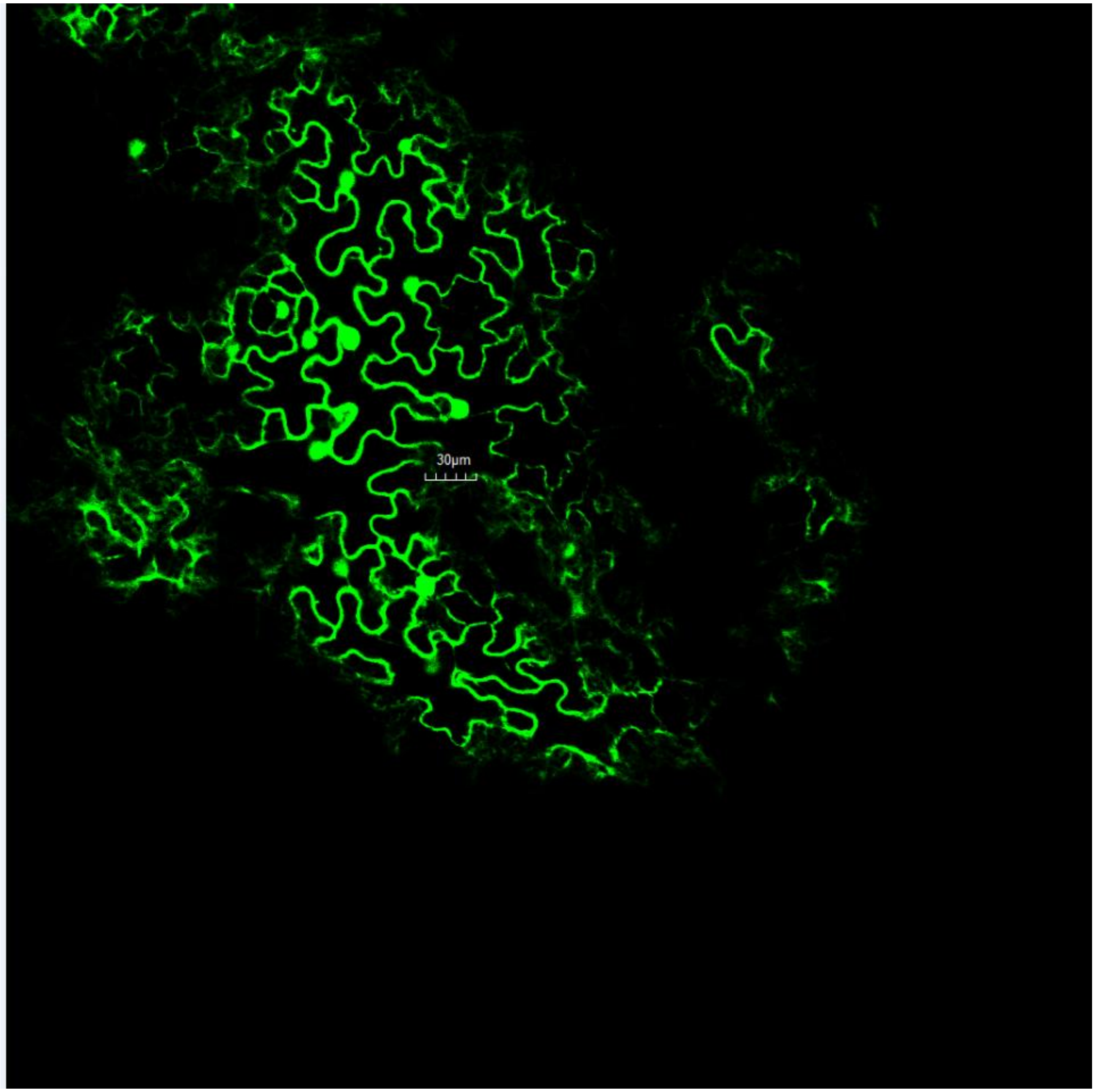

Supplementary Figure 3. The original picture (Subcellular localization of NtCXE22 protein, GFP).

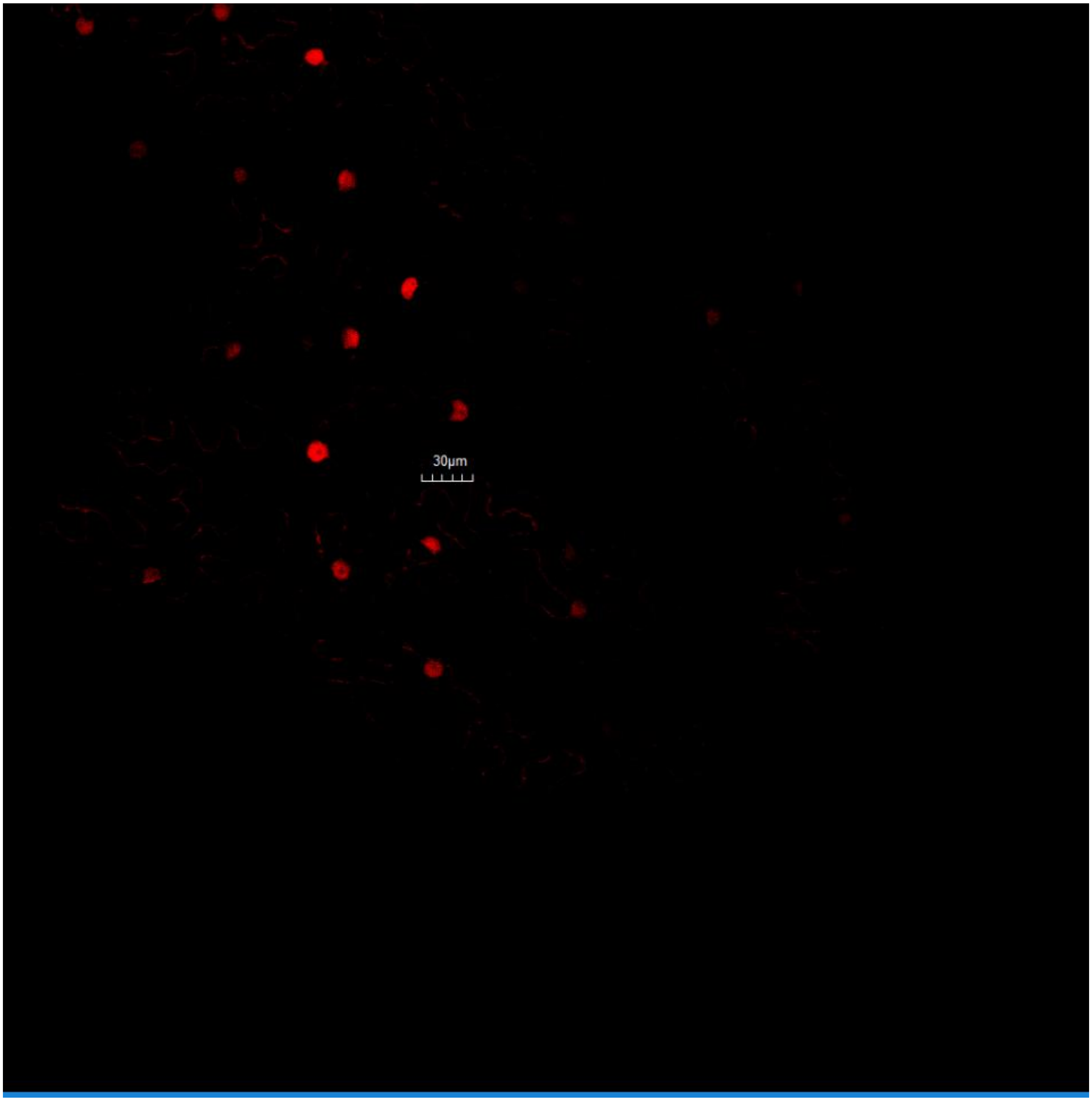

Supplementary Figure 4. The original picture (Subcellular localization of NtCXE22 protein, RFP).

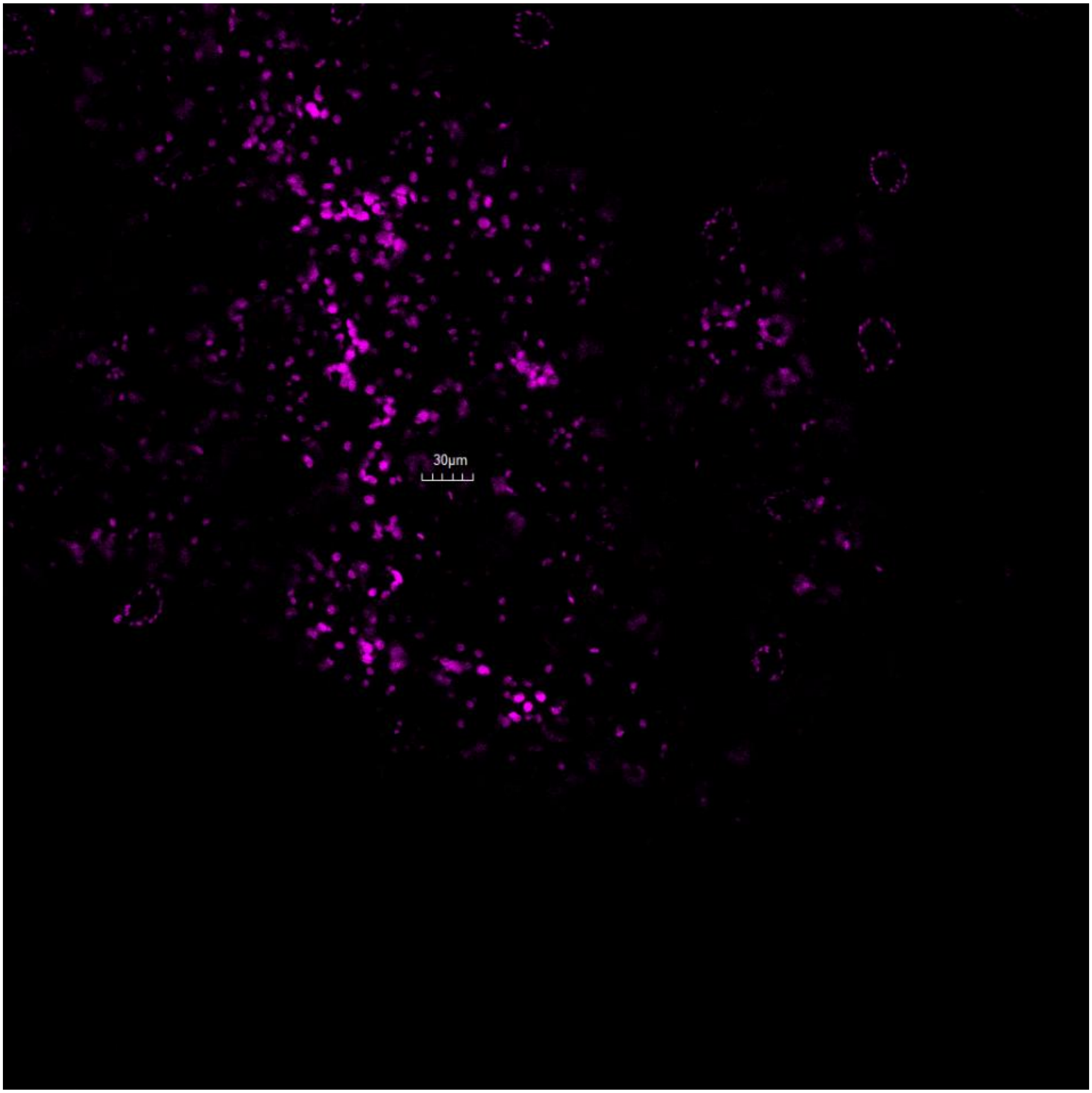

Supplementary Figure 5. The original picture (Subcellular localization of NtCXE22 protein, ChlorophyII fluorescence).

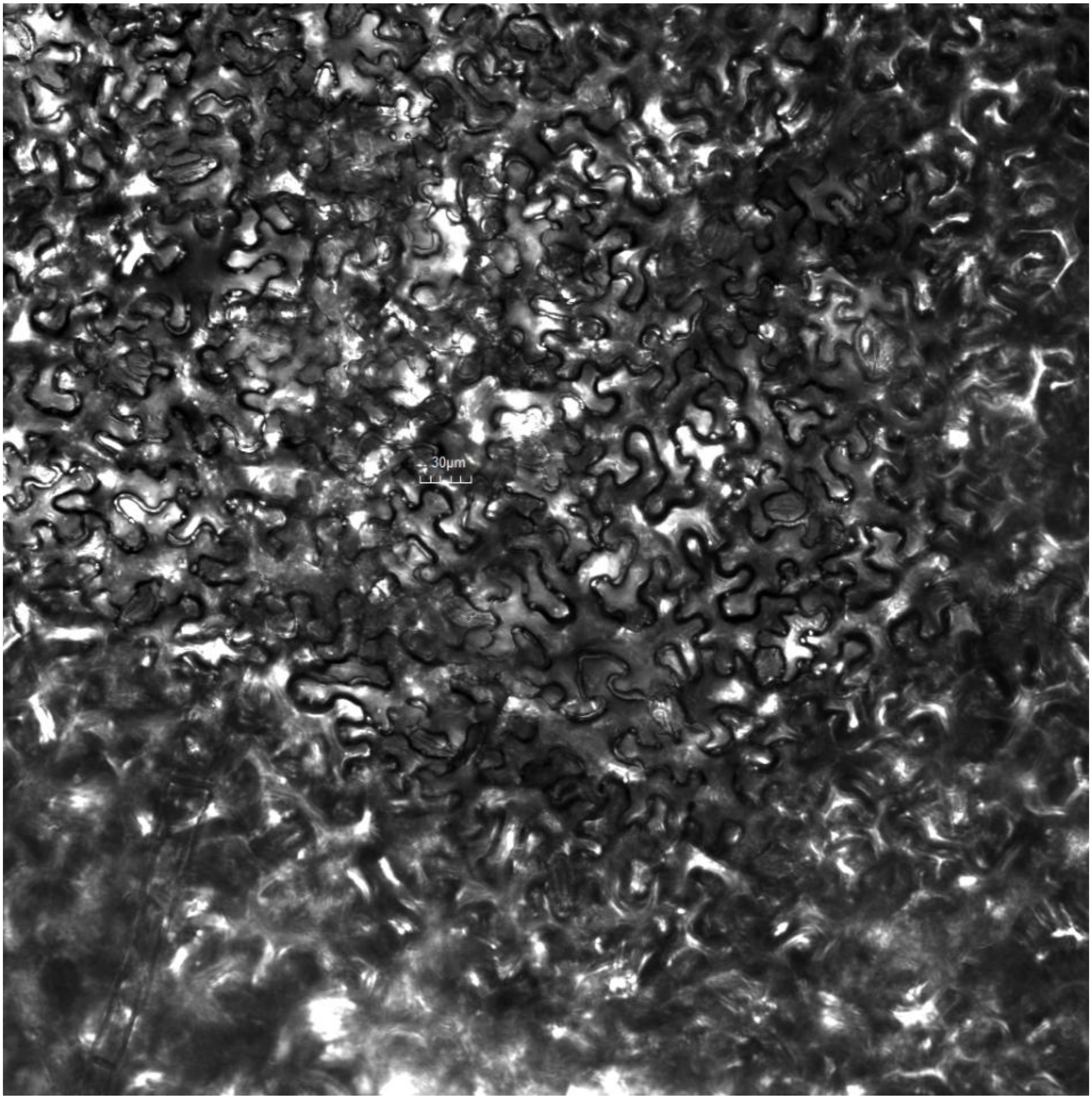

Supplementary Figure 6. The original picture (Subcellular localization of NtCXE22 protein, Bright Field).

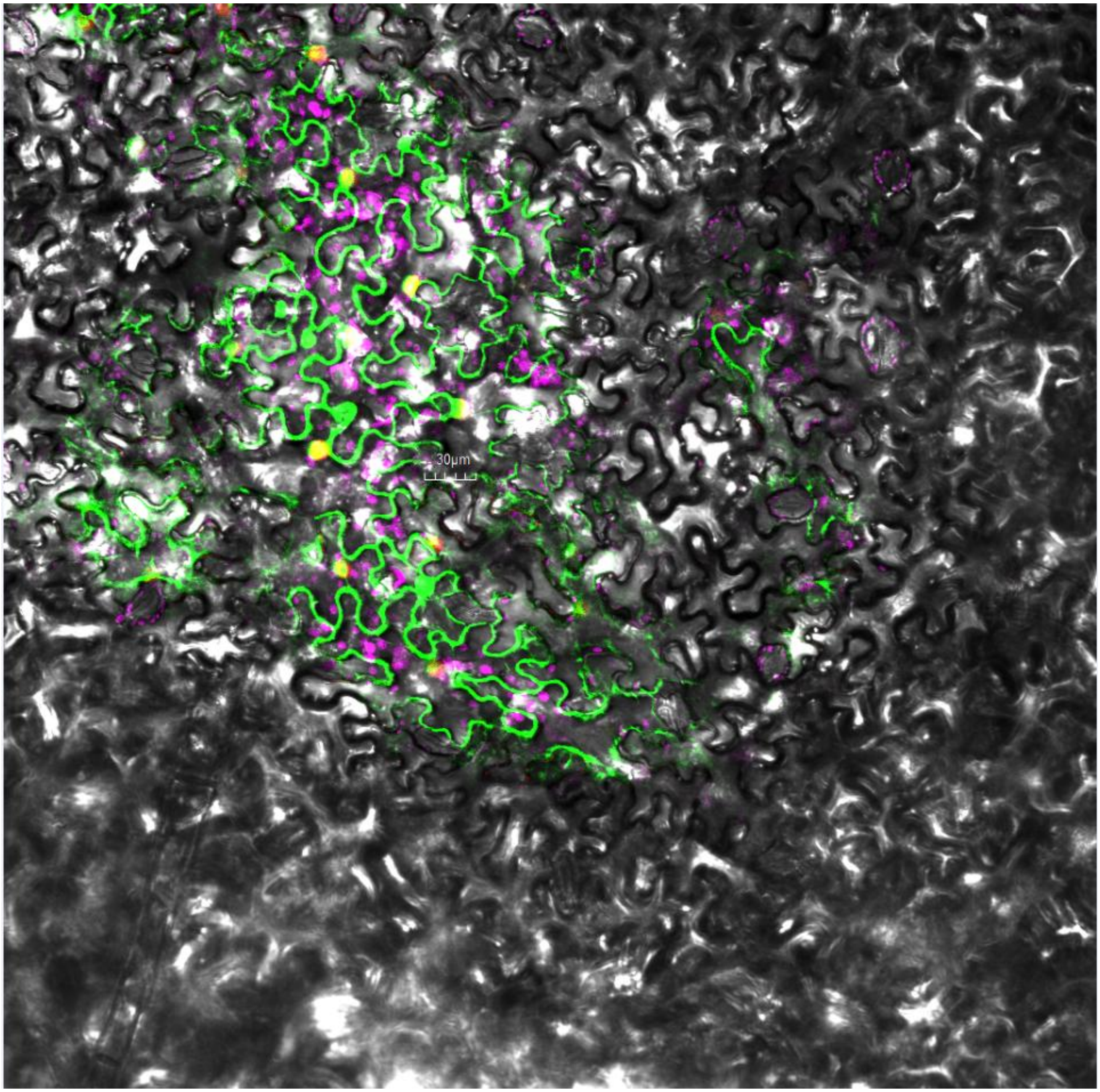

Supplementary Figure 7. The original picture (Subcellular localization of NtCXE22 protein, Merged).

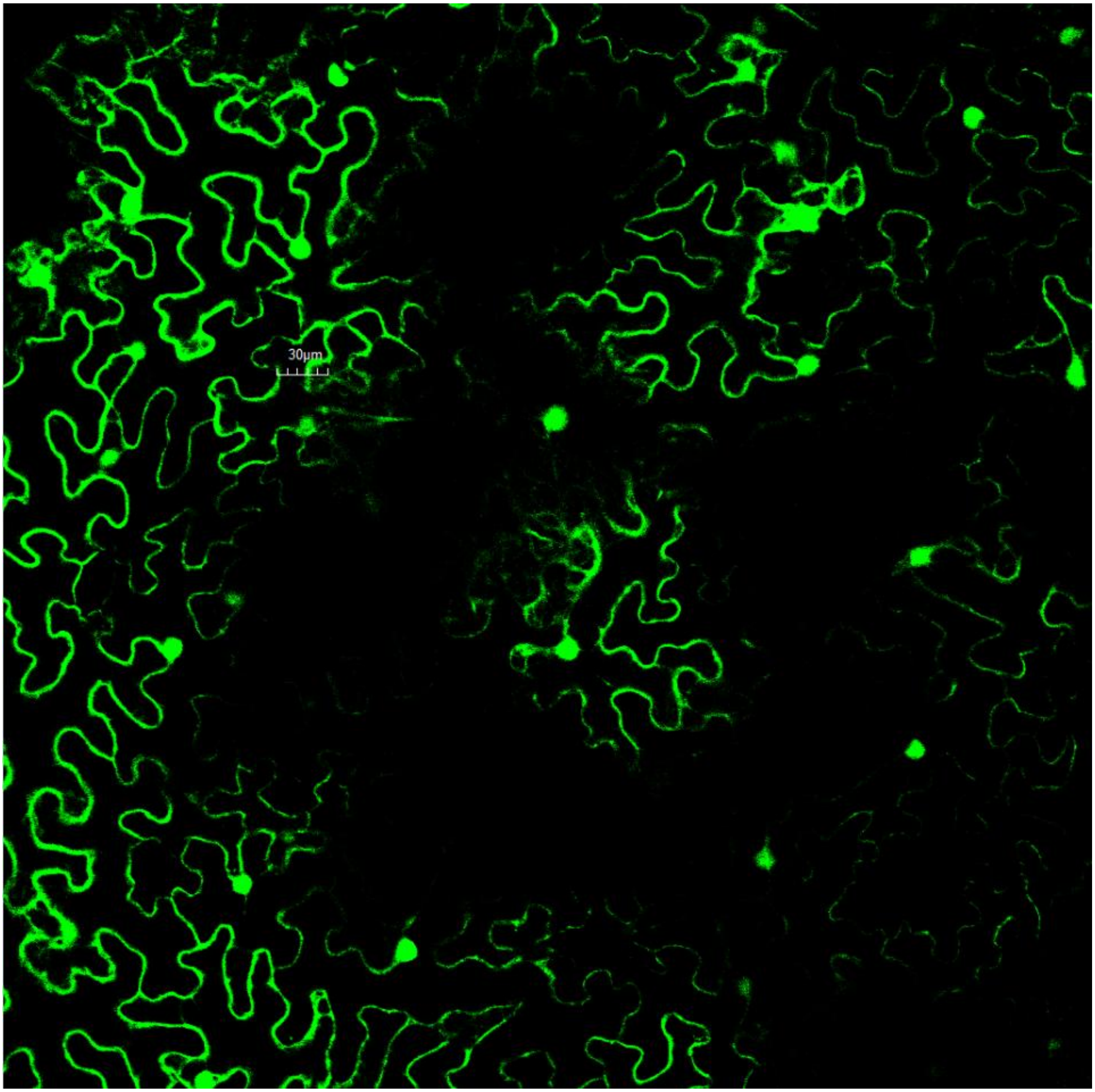

Supplementary Figure 8. The original picture (Subcellular localization of PC1300s-GFP, GFP).

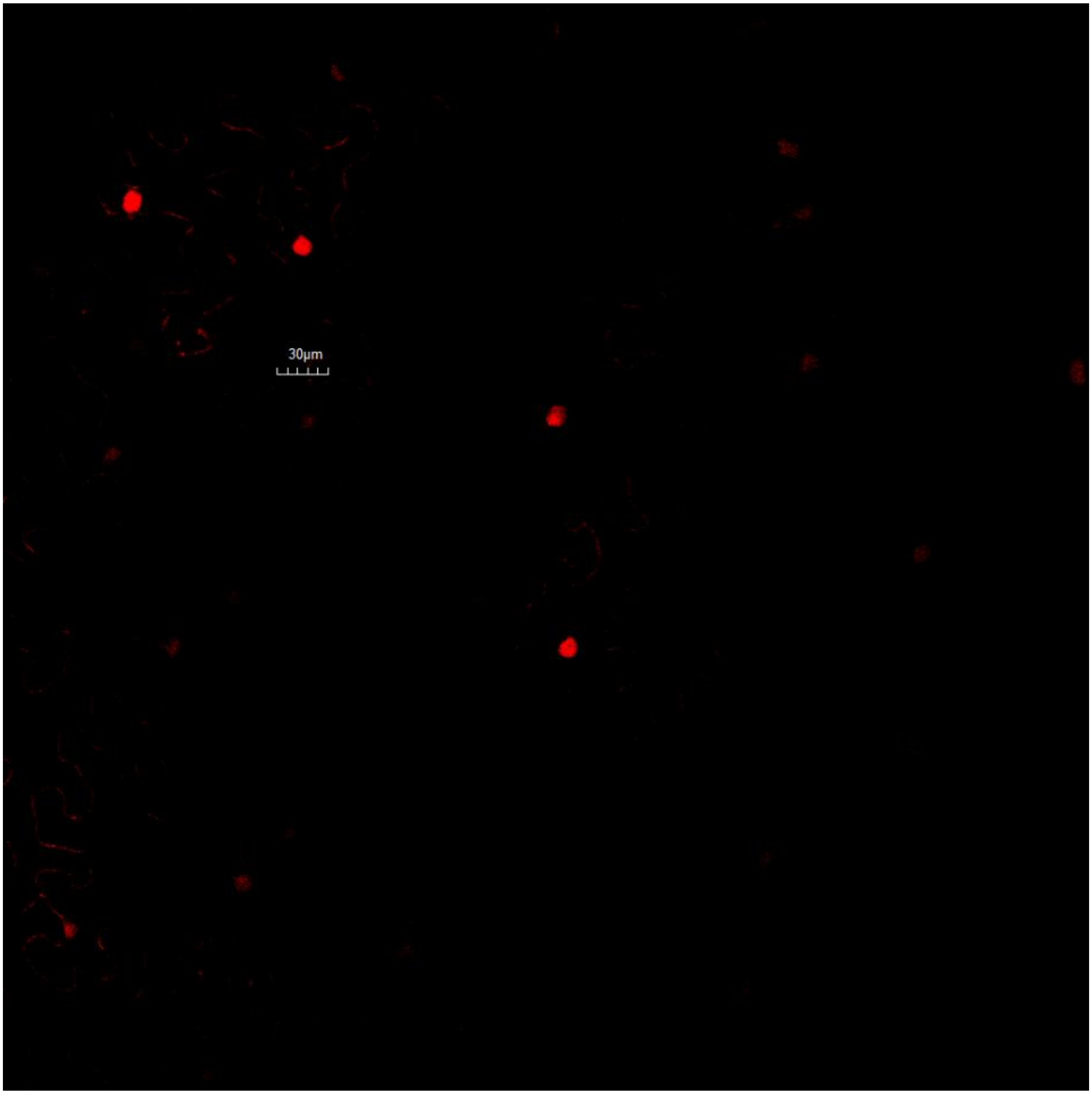

Supplementary Figure 9. The original picture (Subcellular localization of PC1300s-GFP, RFP).

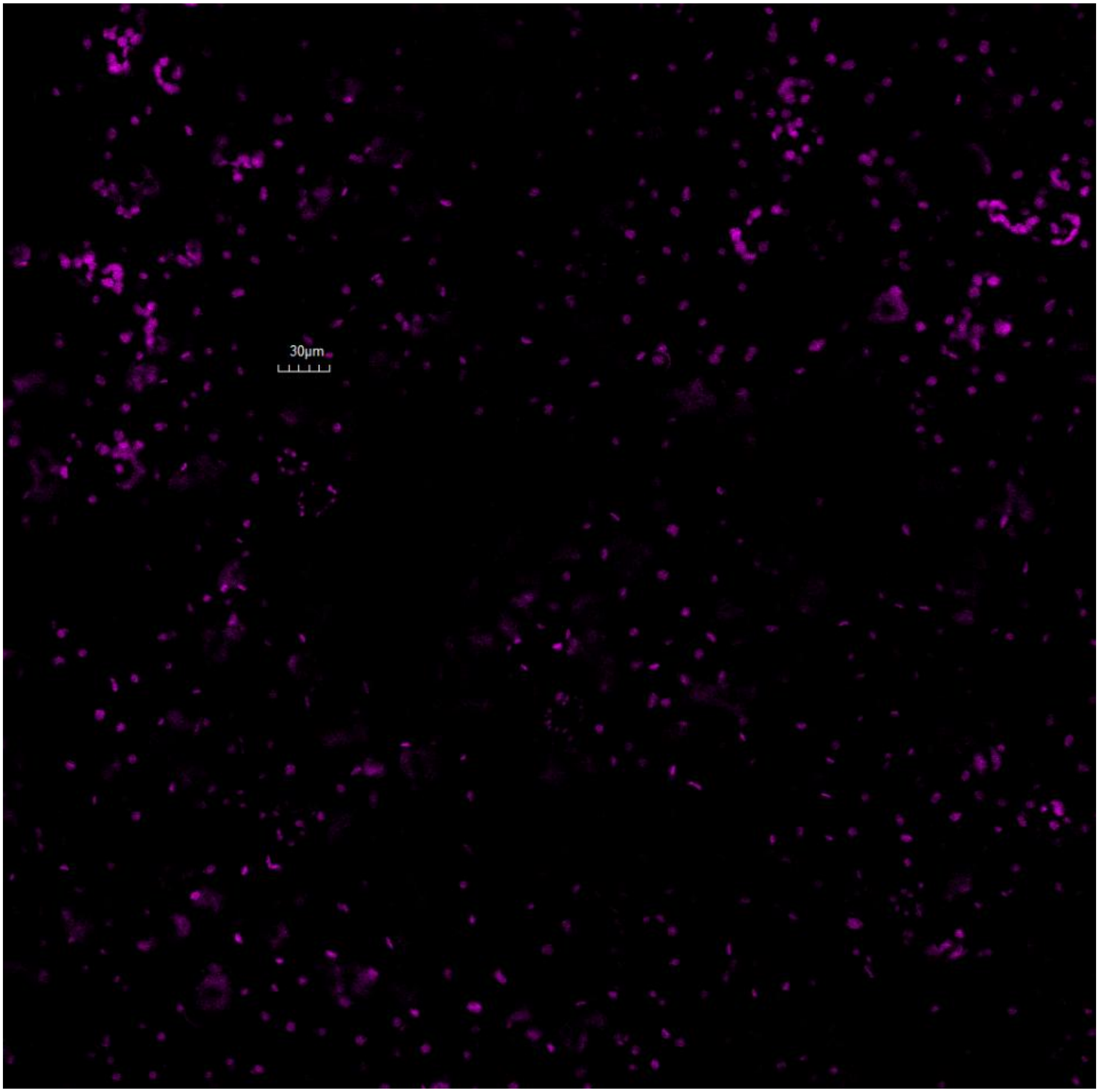

Supplementary Figure 10. The original picture (Subcellular localization of PC1300s-GFP, ChlorophyII fluorescence).

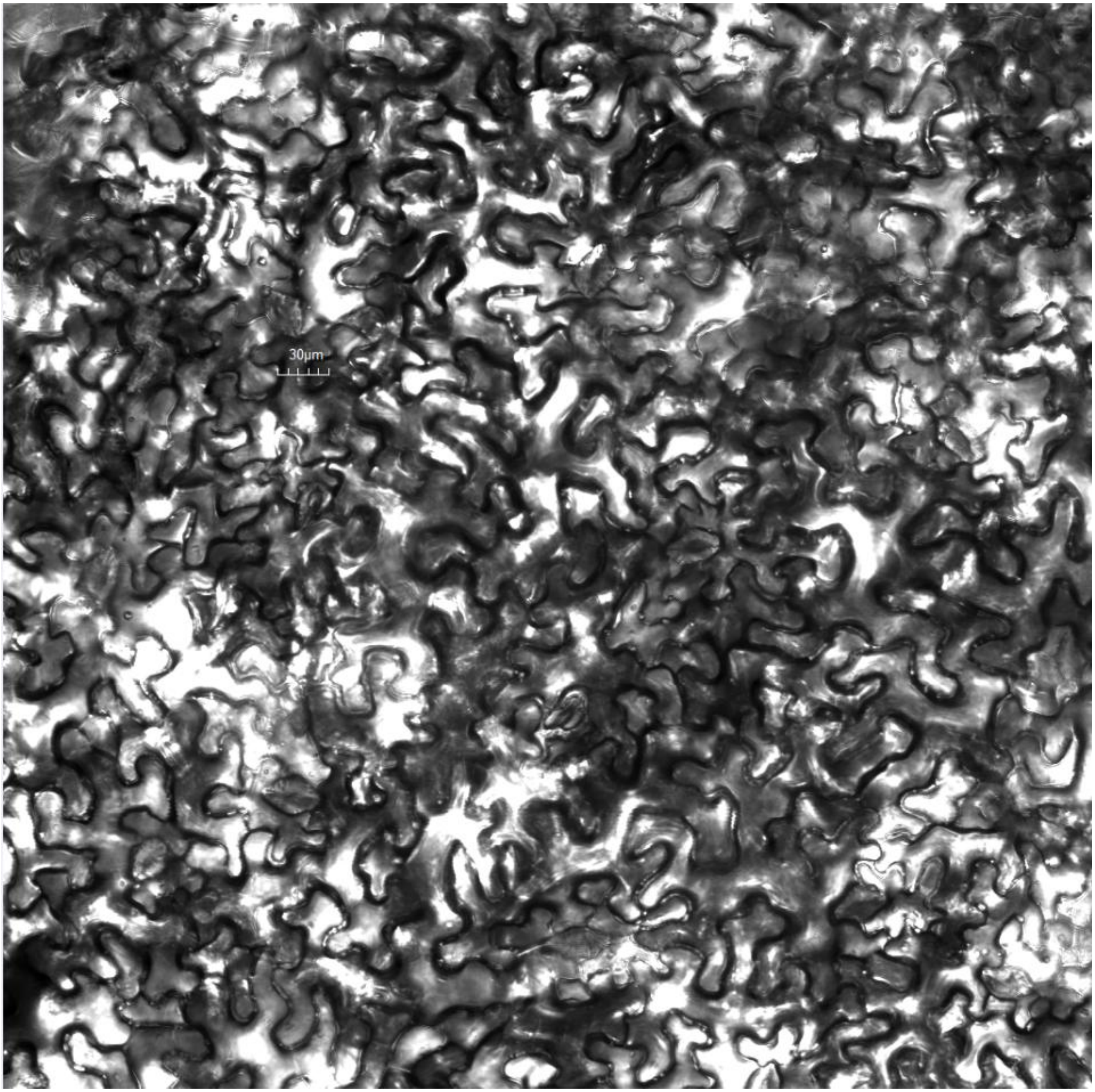

Supplementary Figure 11. The original picture (Subcellular localization of PC1300s-GFP, Bright Field).

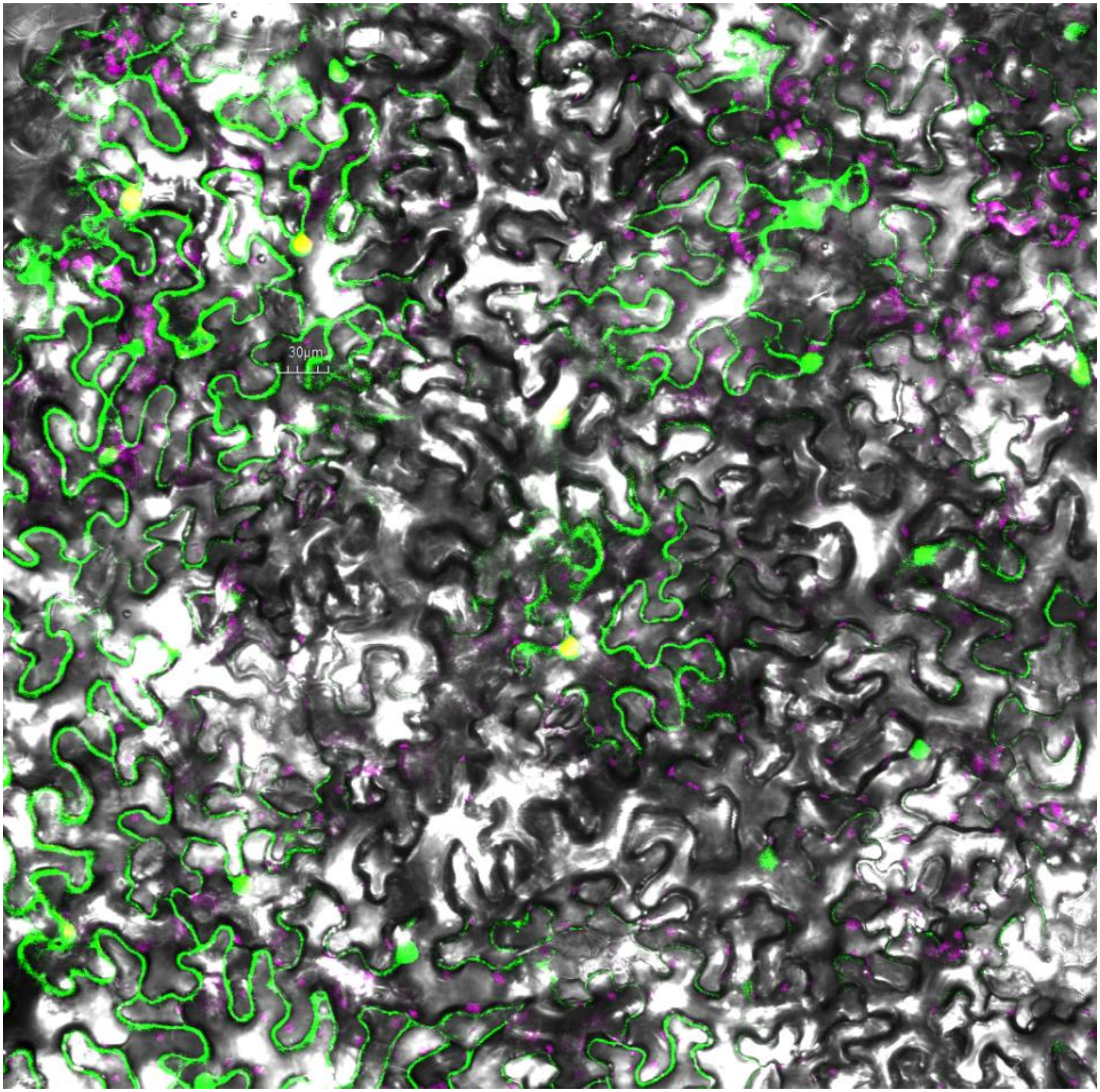

Supplementary Figure 12. The original picture (Subcellular localization of PC1300s-GFP, Merged).

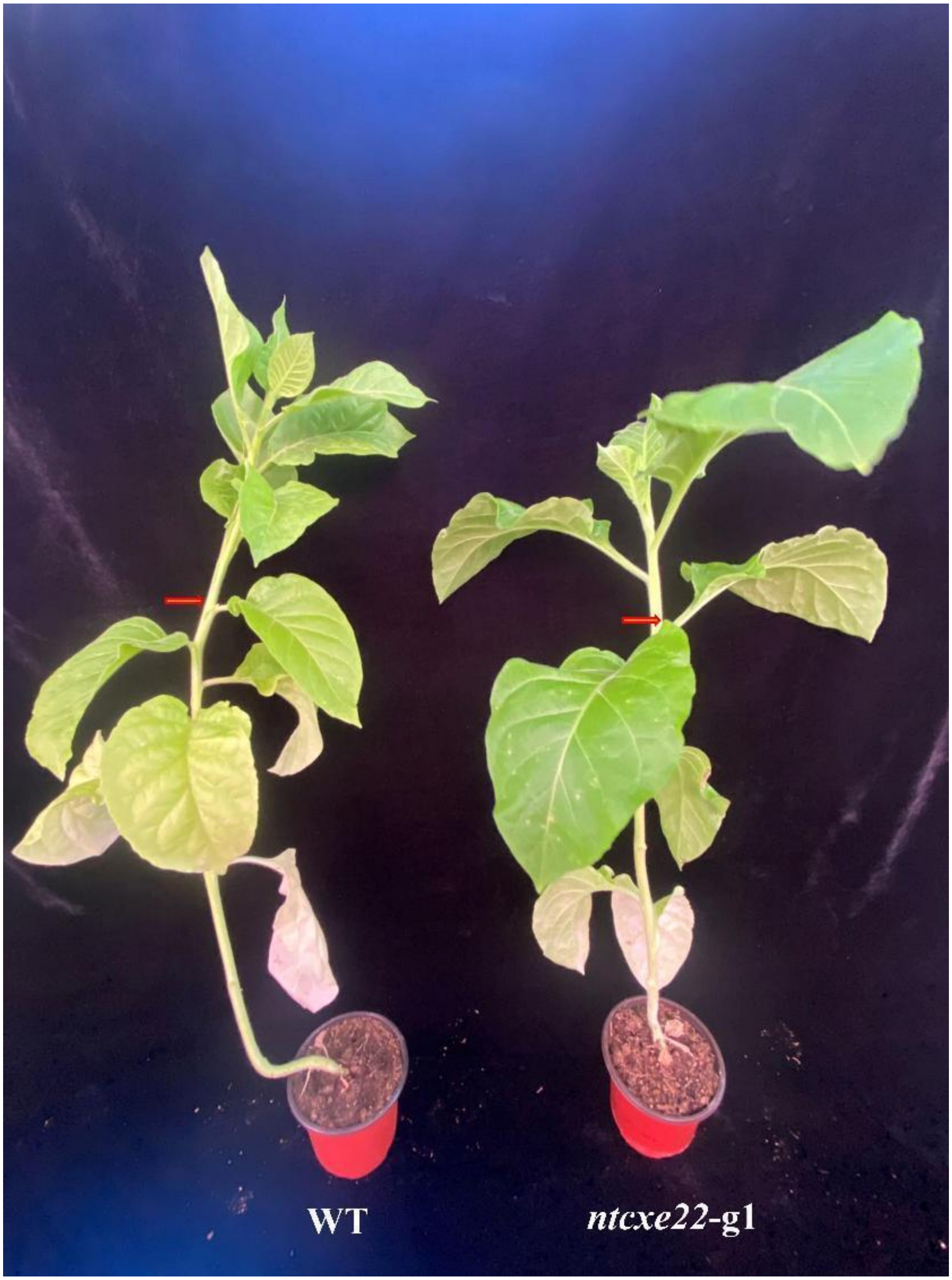

Supplementary Figure 13. The complete plant architecture picture of *ntcxe22*-g1, compared with wild type.

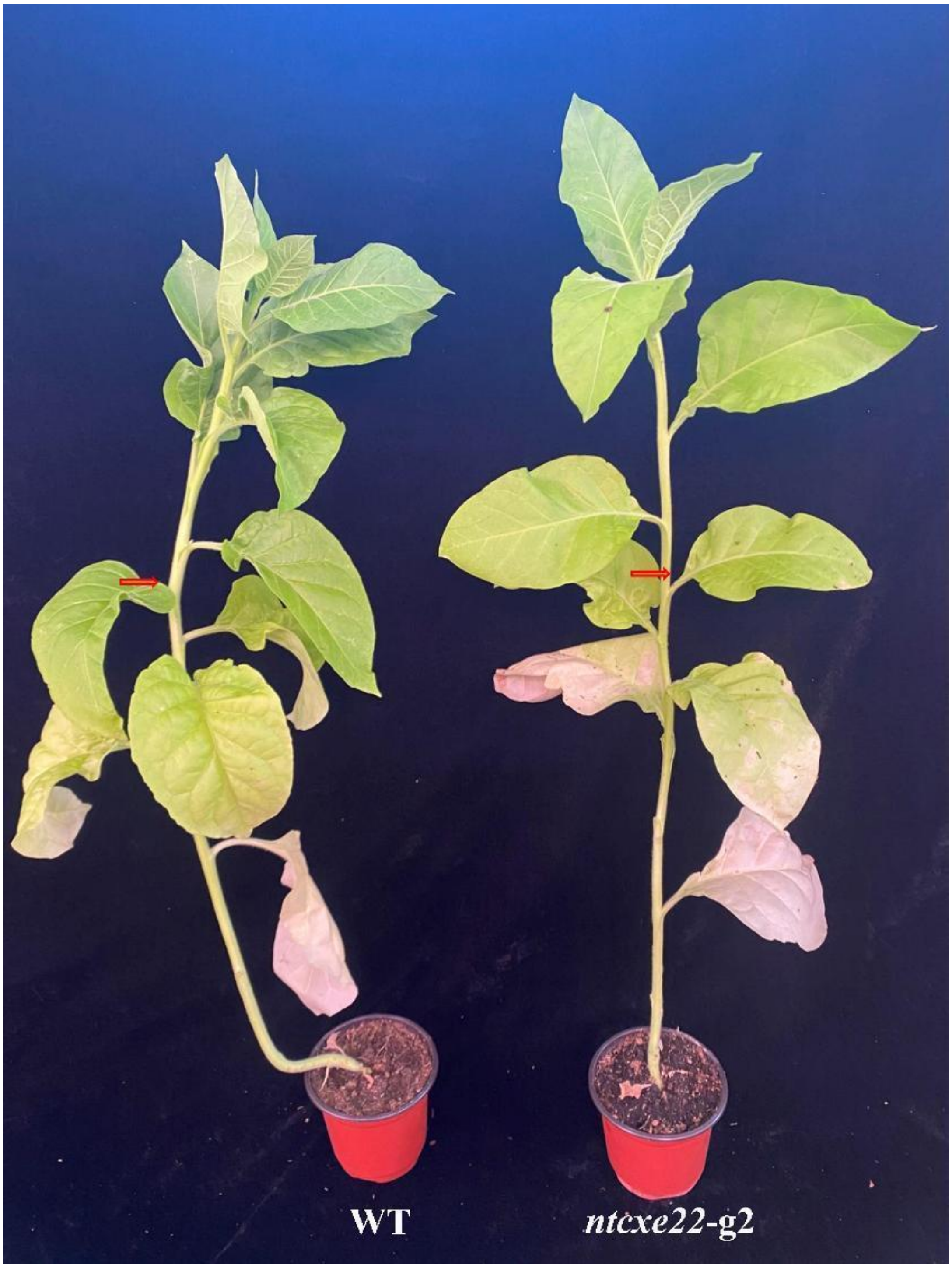

Supplementary Figure 14. The complete plant architecture picture of *ntcxe22*-g2, compared with wild type.

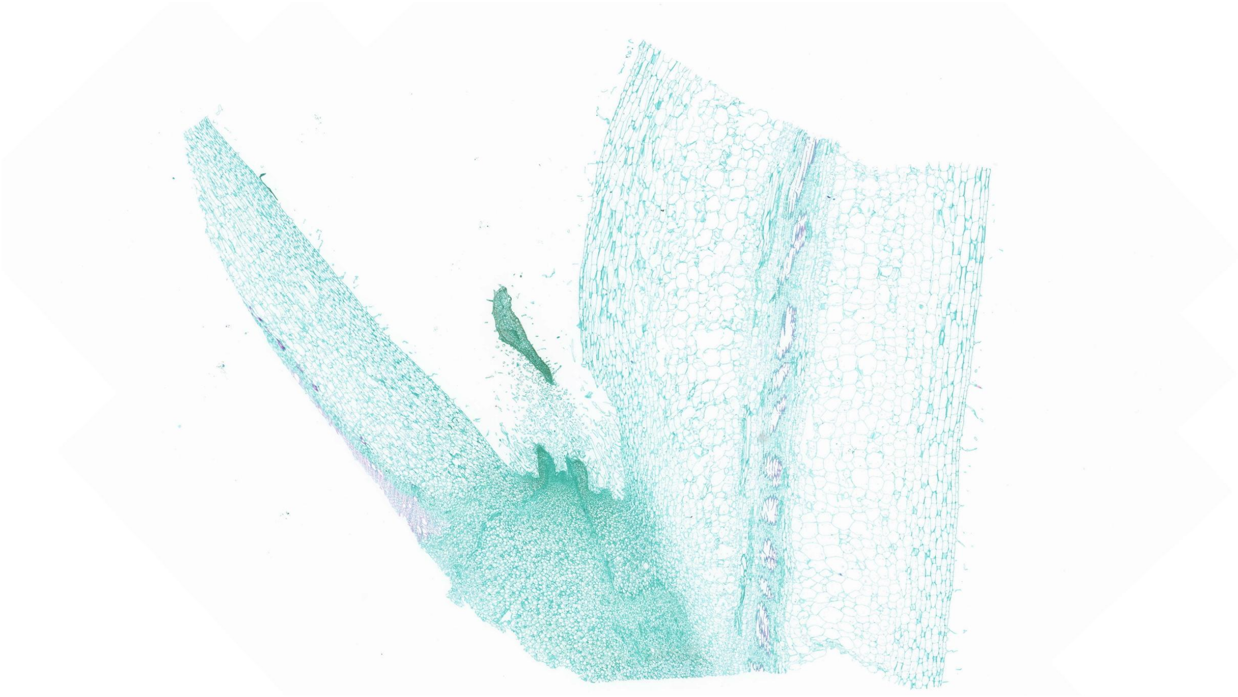

Supplementary Figure15. The original picture (The section staining of axillary bud in wild type).

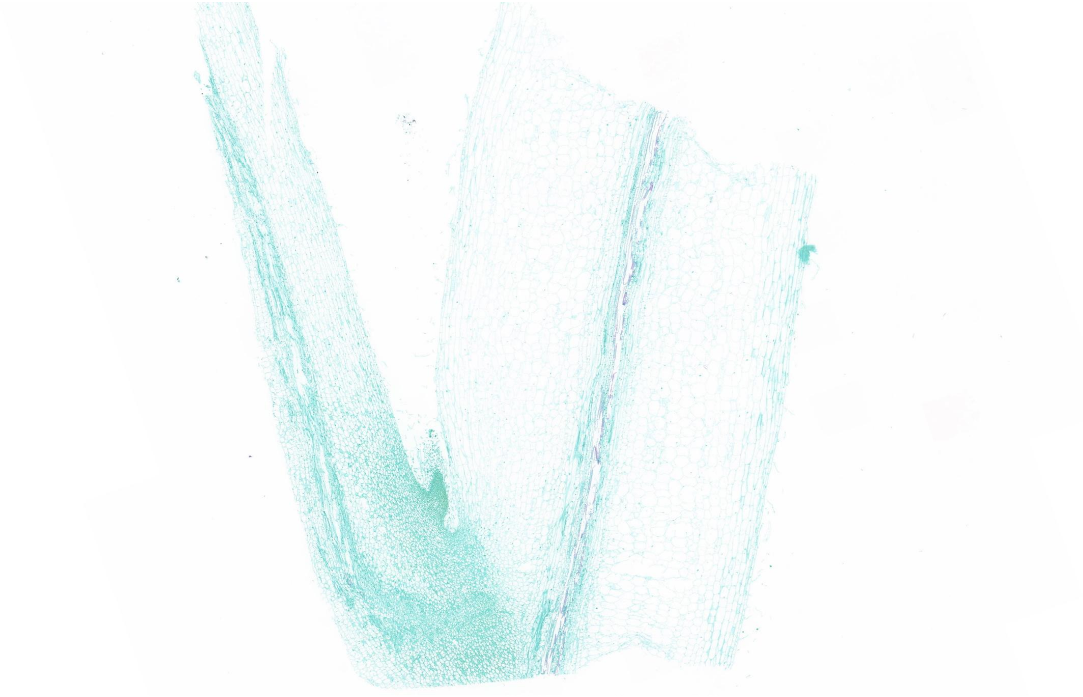

Supplementary Figure 16. The original picture (The section staining of axillary bud in *ntcxe22*-g1).

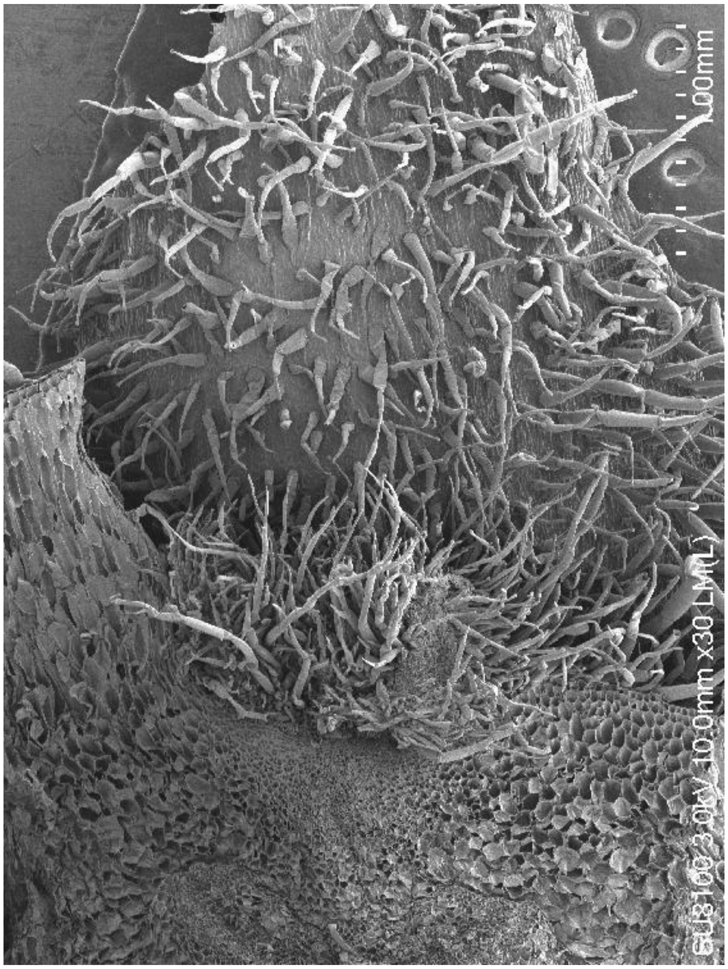

Supplementary Figure 17. The original picture (The scanning electron microscopy of axillary bud in wild type).

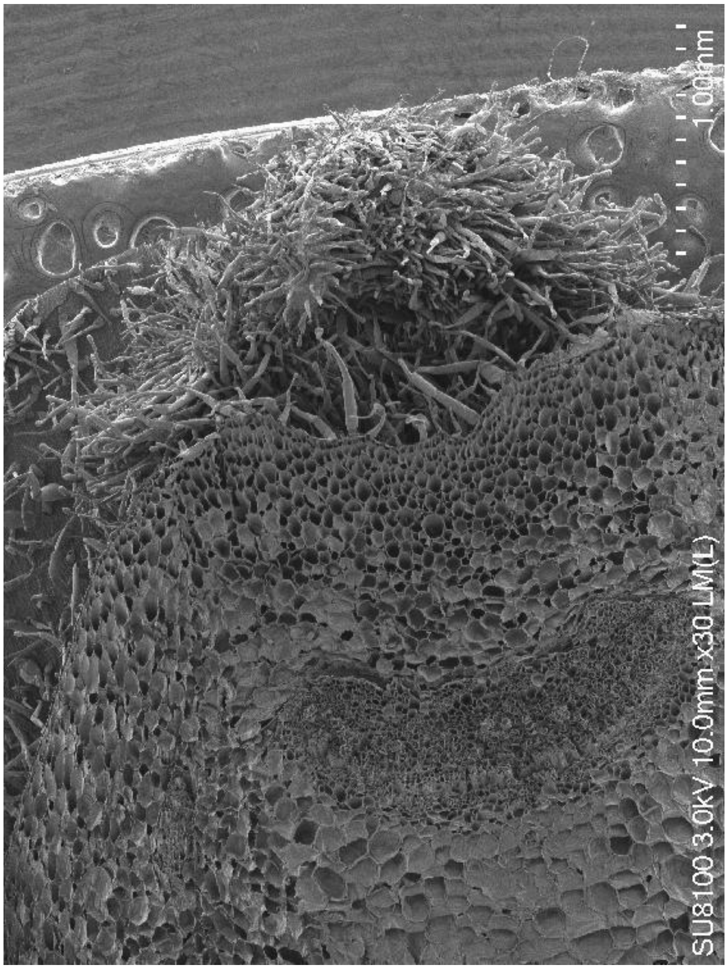

Supplementary Figure 18. The original picture (The scanning electron microscopy of axillary bud in *ntcxe22*-g1).
